# DISC1 Orchestrates Mitochondrial Calcium Overload in Diabetic Encephalopathy Through a Dual Nucleocytoplasmic Mechanism

**DOI:** 10.3390/biom16091366

**Published:** 2026-09-20

**Authors:** Lu He, Hao Li, Rui Yin, Liangli Dai, Weijian Hang, Yong Liu, Man Li, Tao Liang, Yumei Wang, Juan Chen

**Affiliations:** 1 Department of Biochemistry and Molecular Biology, School of Basic Medicine, Tongji Medical College, Huazhong University of Science and Technology, Wuhan 430022, China; 2025tj0059@hust.edu.cn (L.H.); d202582214@hust.edu.cn (R.Y.); d202672271@hust.edu.cn (L.D.); liuyongswe@163.com (Y.L.); 2 Department of Pediatrics, Tongji Hospital, Tongji Medical College, Huazhong University of Science and Technology, Wuhan 430022, China; 3 Key Laboratory of Neurological Diseases of Hubei Province and National Education Ministry, School of Basic Medicine, Tongji Medical College, Huazhong University of Science and Technology, Wuhan 430030, China; d202582217@hust.edu.cn (H.L.); liman73@mails.tjmu.edu.cn (M.L.); 4 Division of Cardiology, Department of Internal Medicine, Tongji Hospital, Tongji Medical College, Huazhong University of Science and Technology, Wuhan 430030, China; hangwjcardio@tjh.tjmu.edu.cn; 5 Department of Clinical Laboratory, Union Hospital, Tongji Medical College, Huazhong University of Science and Technology, Wuhan 430022, China; lt-liangtao-lt@163.com; 6 Department of Nephrology, Union Hospital, Tongji Medical College, Huazhong University of Science and Technology, Wuhan 430022, China; 7 Hubei Key Laboratory of Natural Active Polysaccharides, Union Hospital, Tongji Medical College, Huazhong University of Science and Technology, Wuhan 430022, China

**Keywords:** diabetic encephalopathy, DISC1, mitochondrion, nuclear localization, GRP75

## Abstract

Hyperglycemia-driven mitochondrial dysfunction is a primary driver of diabetic encephalopathy (DE). Here, we identify a novel nucleocytoplasmic “dual effect” of DISC1 that coordinates mitochondrial Ca^2+^ overload under high-glucose conditions. Using nucleocytoplasmic fractionation and mass spectrometry, we demonstrate that high glucose triggers PAK2-mediated phosphorylation of DISC1, necessitating its nuclear translocation. In the nucleus, DISC1 acts as a coactivator for the transcription factor RFX1 to induce Grp75, a critical tethering protein of the GRP75/IP3R1/VDAC1 complex that facilitates Ca^2+^ transfer from the endoplasmic reticulum to mitochondria. Conversely, we find that cytoplasmic DISC1 physically sequesters GRP75, hindering the assembly of the Ca^2+^ conduction complex. Enhanced nuclear translocation of DISC1 results in reduced cytoplasmic DISC1 levels. This depletion removes the “molecular brake” on Ca^2+^ influx, synergizing with the nuclear signaling pathway to drive mitochondrial Ca^2+^ overload. Together, our findings suggest that high glucose hijacks DISC1 through a bipartite mechanism: the upregulation of Ca^2+^ conduction and the concurrent loss of cytoplasmic inhibition. Targeting DISC1 may represent a potential therapeutic strategy for mitigating neurodegeneration in DE.

## 1. Introduction

Diabetic encephalopathy (DE) is a chronic complication of diabetes mellitus characterized by progressive cognitive decline, impaired synaptic plasticity, and structural brain atrophy [1,2]. The rising global diabetic population has elevated DE to a prominent public health concern, significantly affecting the quality of life for numerous individuals [3]. Despite its widespread clinical presence, the precise molecular progression from systemic metabolic dysfunction to localized neuronal injury remains incompletely understood. Therefore, it is imperative to elucidate the mechanisms underlying diabetic neuronal damage and identify novel therapeutic targets to hinder its progression.

Recent studies emphasize the critical involvement of mitochondrial dysfunction in the pathophysiology of DE [4,5]. Mitochondria are essential for meeting the high metabolic demands of neurons [6,7], yet they are highly susceptible to the systemic “fuel overload” of diabetes. The physical and functional interface between the ER and mitochondria, known as the Mitochondria-Associated Membrane (MAM) [8,9], facilitates this ion transfer via a specialized macromolecular bridge composed of Inositol 1,4,5-trisphosphate receptor (IP3R), the chaperone Glucose-Regulated Protein 75 (GRP75), and Voltage-Dependent Anion Channel 1 (VDAC1) [10]. Specifically, mitochondrial Ca^2+^ overload has been identified as a critical executioner of neuronal apoptosis [11]. Basal Ca^2+^ levels are essential for activating the tricarboxylic acid (TCA) cycle and facilitating ATP synthesis [12]. However, an excessive influx of Ca^2+^ induces the opening of the mitochondrial permeability transition pore, resulting in the release of cytochrome c and subsequent cell death [13]. The main intracellular Ca^2+^ reservoir is the endoplasmic reticulum (ER), which is the primary origin of this excess Ca^2+^ [8,9]. Hyperglycemia has been shown to enhance Ca^2+^ transport through MAMs, resulting in mitochondrial Ca^2+^ overload [14]. However, the mechanisms that inhibit this aberrant flux, and their dysfunction under diabetic conditions, are not fully elucidated.

Disrupted in Schizophrenia 1 (DISC1) functions as a versatile scaffold protein, crucial for maintaining mitochondrial homeostasis by facilitating trafficking and mitophagy [15,16]. While historically linked to psychiatric disorders [17,18], recent research suggests that DISC1 also plays a protective role in high-energy-demand tissues affected by conditions such as myocardial infarction, Alzheimer’s disease, and metabolic disorders [19,20,21]. DISC1 has also been shown to prevent mitochondrial Ca^2+^ overload by interacting with IP3R and disrupting the GRP75-mediated ER-mitochondria channel coupling [22]. The mechanism through which hyperglycemia utilizes DISC1 signaling to induce Ca^2+^ overload in the diabetic brain remains unclear. Furthermore, the spatial regulation of DISC1 remains poorly understood. Despite possessing a nuclear localization sequence (NLS) and exhibiting nucleocytoplasmic shuttling [23], the precise mechanisms governing its translocation and transcriptional activity, particularly the impact of phosphorylation, remain ambiguous [24].

In this study, we demonstrate that hyperglycemia induces diabetic encephalopathy by regulating a novel nucleocytoplasmic mechanism involving DISC1, which coordinates mitochondrial Ca^2+^ overload. High glucose stimulates p21 (RAC1)-activated kinase 2 (PAK2)-mediated phosphorylation of DISC1, facilitating its nuclear translocation. In the nucleus, DISC1 interacts with the transcription factor regulatory factor X1 (RFX1) to upregulate *Grp75* expression, enhancing ER-mitochondrial Ca^2+^ transfer. Simultaneously, the nuclear translocation of DISC1 leads to a reduction in cytoplasmic DISC1 levels. This reduction eliminates the inhibitory effect of DISC1 on Ca^2+^ influx, promoting the formation of the conduction complex. Through this coordinated mechanism in both the nucleus and cytoplasm, hyperglycemia utilizes DISC1 to induce detrimental mitochondrial Ca^2+^ overload and neuronal damage.

## 2. Materials and Methods

### 2.1. Diabetic Animal Models

All animal procedures were approved by the Committee of the Experimental Animal Centre of Servicebio under the ethical approval No. 2022233. Eight-week-old db/db and littermate wild-type control mice were purchased from Beijing Vital River Laboratory Animal Technology Co., Ltd. (Beijing, China). Wild-type male C57BL/6 mice were also purchased from Beijing Vital River Laboratory Animal Technology Co., Ltd. (Beijing, China). These mice underwent a 12 h light/dark cycle with free access to water and food in the SPF Experimental Animal Center at Tongji Medical College of Huazhong University of Science and Technology. Mice were sacrificed at 24 weeks of age.

Group allocation, blinding and exclusion criteria. A total of 10 db/db mice and 7 wild-type littermates were used. Mice of both sexes were included and sex was balanced across groups. Animals were assigned to experimental groups (simple randomisation using a computer-generated random number sequence). Investigators responsible for behavioural testing, image acquisition and quantitative analysis were blinded to genotype and treatment until the analysis was completed. Three db/db mice died prematurely and were therefore excluded.

Body weight, serum triglyceride, total cholesterol, and glucose were measured at week 24. Serum triglyceride, total cholesterol, and glucose were measured via a triglyceride kit (Nanjing Jiancheng Bioengineering Institute, A110-1-1, Nanjing, China), total cholesterol (Nanjing Jiancheng Bioengineering Institute, A111-1-1, Nanjing, China), and glucose colorimetric assay kit (Beyotime, S0202S, Shanghai, China).

### 2.2. Brain Stereotactic Injection

Stereotaxic injection was performed using bregma as the reference point [25]. The sagittal suture was taken as the Y-axis, and the vertical axis passing through bregma was defined as the X-axis. AAV9 carrying an shRNA targeting *Rfx1* under the control of the hSyn promoter (titer: 1 × 10^10^ Tu/mL) was injected into the CA1 region of the mouse hippocampus at coordinates X: 2 mm, Y: 2.5 mm, Z: 1.7 mm. A volume of 300 nl was delivered unilaterally at an infusion rate of 15 nl/min. The animals were allowed to recover for 1–2 weeks before subsequent experiments. All injections, including those of the control virus, were delivered unilaterally into the CA1 region of the right hippocampus. The control virus (AAV9-hSyn-shNC-EGFP) was injected at identical coordinates, volume and infusion rate. Stereotaxic surgery was performed at week 8; behavioural testing was carried out 16 weeks later; and animals were sacrificed at week 24 for molecular and histological analyses.

### 2.3. HT22, BV2, C8-D1A, SH-SY5Y Cell Culture

The HT22 cell line (Servicebio, STCC20011P, Wuhan, China), BV2 cell line (Servicebio, STCC20009P, Wuhan, China), C8-D1A cell line (Procell, CL-0506, Wuhan, China) was cultured in DMEM (Gibco, 11995065, Grand Island, NY, USA) supplemented with 10% fetal bovine serum (Yeasen, 40131ES76, Shanghai, China) and maintained in a constant temperature incubator at 37 °C with 95% humidity and 5% CO_2_. The SH-SY5Y human neuroblastoma cell line (Servicebio, STCC11004P, Wuhan, China) was cultured in DMEM/F12 (Procell, PM150312, Wuhan, China) supplemented with 10% fetal bovine serum and maintained at 37 °C in a humidified atmosphere containing 5% CO_2_.

### 2.4. Primary Mouse Neuron Culture

Primary neurons were prepared from C57BL/6 mouse embryos at E18. Tissue was dissociated in trypsin (BioFROXX, 1004GR025, Offenbach am Main, Germany) for 5 min at 37 °C and plated onto poly-L-lysine-coated dishes. Cells were maintained in Neurobasal medium (Beyotime, C2781, Shanghai, China) supplemented with 2% B27 (Beyotime, C0347, Shanghai, China), 0.5 mM GlutaMAX and 1% penicillin–streptomycin, with half of the medium replaced every 2 days. Neuronal identity was confirmed by MAP2 immunostaining.

### 2.5. High-Glucose Treatment and Osmotic Control

According to previous reports [26], D-glucose (MCE, HY-B0389, Monmouth Junction, NJ, USA) was added to give a final concentration of 50 mM. In all cell experiments involving high-glucose treatment, cells in the non-high-glucose control group were exposed to medium supplemented with mannitol at an equimolar concentration, which matches the osmotic load of the high-glucose condition without providing additional metabolizable glucose. The mannitol osmotic-control data are presented in Figures 2–4, 6, 8 and Figures S1–S3 and S5.

### 2.6. RNA Extraction and Real-Time PCR

Cell RNA was extracted using TRIzol (Vazyme) and reverse-transcribed into cDNA using oligo (dT) primers (Vazyme). Total cell cDNA was used as the template for real-time PCR, and real-time PCR was performed using ChamQ SYBR qPCR Master Mix (Vazyme) with the following primers on a CFX96. Relative mRNA level was quantified by the 2^−ΔΔCt^ method. The primer sequences used are listed below (5′-3′): *Mcur1* AAGATGCAGCAGGAAATCGC/ TGTCCGGACTTTGGTCACTT, *Akt1* GCCGCCTGATCAAGTTCTCC/ TTCAGATGATCCATGCGGGG, *Smdt1* AGAACTTCGCTGCTCTGCTT/ GCTCCCTGTGCCCTGTTAAT, *Afg3l2* GATCGCCCGCCTCGG/ AGCATATTGATAGAGCGTACGGA, *Itpr1* CAAGCAACTGCTGGAGGAGA/ TTCAAGCTCCTGCTCTGTGG, Mcu GCCACCAAAGAGAGACCTCC/ GCTCAATGCACAGTGTGGTG, *Spg7* GGGTCACCTCTGGAAGCAAA/ AGGTTGTCTAGCAGCACCTTC, Disc1 TAGGCGTGAAGCTCCTACAC/ AGAGAGCCTCATCGTGACTGT, *Actin* GGCTGTATTCCCCTCCATCG/ CCAGTTGGTAACAATGCCATGT.

### 2.7. Mass Spectrometry (MS) Analysis

Protein digestion and mass spectrometry were conducted by Beijing Novogene Technology Co., Ltd. (Beijing, China). The protein-containing gel obtained by Western blot was stained with Coomassie brilliant blue. Target protein bands were then excised from the gel and submitted to Beijing Novogene Corporation for MS analysis.

### 2.8. siRNA Transfection

The siRNA was synthesized by Wuhan Jinkairei Biotechnology Co., Ltd. (Wuhan, China). Cells were seeded in appropriate culture plates to reach 50% confluence at the time of transfection. siRNA was transfected using non-liposomal PEI transfection reagent (Yeasen, 40806ES, Wuhan, China) according to the manufacturer’s instructions. Transfection efficiency was assessed 48 h post-transfection using 50 nM of control siRNA (NC) or *Grp75*-targeted siRNA (siGrp75) (sense, 5′-GCGUCUUUACCAAACUUAUTT-3′; antisense, 5′- AUAAGUUUGGUAAAGACGCTT-3′), or *Disc1*-targeted siRNA (siDisc1) (sense, 5′- GAGACUUGUCAGUUGCUAATT-3′; antisense, 5′- UUAGCAACUGACAAGUCUCTT-3′), or *Rfx1*-targeted siRNA (siRfx1) (sense, 5′- GCAGGCACCUACGUGAUCCAATT-3′; antisense, 5′- UUGGAUCACGUAGGUGCCUGCTT-3′), or *Pak2*-targeted siRNA (siPak2) (sense, 5′- UUCUCCGAACGUCACGUTT-3′; antisense, 5′- ACGUGACACGUUCGGAGAATT-3′).

### 2.9. Plasmid DNA Construction and Transfection

pcDNA3.1-DISC1-Flag was cloned into the pcDNA3.1(+)-Flag vector. pcDNA3.1-DISC1-△NLS-Flag was cloned into the pcDNA3.1(+)-Flag vector. pcDNA3.1-RFX1-Flag was cloned into the pcDNA3.1(+)-Flag vector. pcDNA3.1-LEF1-Flag was cloned into the pcDNA3.1(+)-Flag vector. pcDNA3.1(+)-GRP75-Flag was cloned into the pcDNA3.1(+)-Flag vector. pcDNA3.1(+)-DISC1^S284E^-Flag was cloned into the pcDNA3.1(+)-Flag vector. pcDNA3.1(+)-DISC1^S285E^-Flag was cloned into the pcDNA3.1(+)-Flag vector. pcDNA3.1(+)-DISC1^S285A^-Flag was cloned into the pcDNA3.1(+)-Flag vector. All constructions were confirmed by DNA sequencing.

According to the manufacturer’s instructions, plasmid DNA was transfected into cells using Polyethylenimine Linear (40816ES01, Yeasen, Shanghai, China).

### 2.10. In Vitro Kinase Assay

For in vitro kinase assays, recombinant active PAK2 was incubated with purified wild-type DISC1 or the DISC1 S285A mutant in reaction buffer containing 50 mM Tris-HCl (pH 7.5), 10 mM MgCl_2_, 1 mM DTT, and 100 µM ATP at 30 °C for 30 min. Parallel reactions without PAK2 or without ATP were performed as negative controls. The reactions were terminated by adding 5× SDS-PAGE loading buffer and boiling at 95 °C for 5 min. Phosphorylation of DISC1 was analyzed by a phospho-specific antibody.

### 2.11. Ultrastructure Observation by Transmission Electron Microscopy

After fixing the cell or tissue in 2.5% glutaraldehyde at room temperature for 2 h and at 4 °C for 24 h, the fixed samples were embedded, and ultrathin sections were prepared by ServiceBio in Wuhan. After proper staining, an ultrathin section was observed using a Hitachi transmission electron microscope.

After TEM imaging, the images were analyzed in ImageJ 1.53t using specific plugins. Mitochondrial morphology was analyzed using MiNA 3.0.0.

### 2.12. Cell Proliferation Assay

Cell proliferation was measured by the CCK8 assay (KTA1020, Abbkine, Wuhan, China). Briefly, 4000 cells were seeded into each well of a 96-well plate. Following cell treatment, the optical density of the different groups was measured by adding 10% CCK-8 solution to the culture medium and incubating at 37 °C for 30 min. Wells containing only 10% CCK-8 solution without cells were designated as blank controls.

### 2.13. Nucleocytoplasmic Fractionation

Nuclear and cytoplasmic fractions were prepared using a commercial kit (P0028, Beyotime, Shanghai, China) according to the manufacturer’s instructions. Fraction purity was assessed in every experiment by immunoblotting for H3 and β-tubulin.

### 2.14. Chromatin Immunoprecipitation Combined with qPCR (ChIP-qPCR)

ChIP assays were conducted according to the protocol provided with a commercial kit (RK20258, Abclonal, Wuhan, China). An anti-RFX1 antibody (26859-1-AP, Proteintech, Wuhan, China) and an anti-DISC1 antibody (A4678, Abclonal, Wuhan, China) were used for immunoprecipitation to capture DNA fragments. Following DNA purification with a commercial extraction kit (RK30100, Abclonal, Wuhan, China), the samples were subjected to qPCR to evaluate the enrichment of Rfx1 at the *Grp75* promoter.

The following primer pair was employed for qPCR:

*Grp75* forward, AAGAACCAACACCCCACGG; reverse, GCGGACACGTAGTCTCTAGTC.

*GRP75* forward, GTACGAGGCAGCAAACAAGC; reverse, CGAGTATCCTCTGGTCAGGC.

### 2.15. Protein Extraction and Western Blotting

Protein extraction and Western blotting were performed as previously described [27]. Briefly, after the indicated stimulations, cells or animal samples were harvested and lysed with RIPA. After incubating on ice for 15 min, the lysate was centrifuged at 16,000× *g* at 4 °C for 15 min, and the supernatant was collected into a new pre-cooled Eppendorf tube. Protein concentration was quantified by the BCA method. Proteins were separated by SDS-PAGE and transferred to a PVDF membrane (0.45 μm). After blocking the membrane with 5% BSA, the membrane was incubated with the intended primary antibody overnight at 4 °C. HRP-conjugated secondary antibody and ECL were used to detect the protein bands.

The primary antibodies used were as follows: anti-DISC1 (A4678, Abclonal, Wuhan, China), anti-ACTIN (AC026, Abclonal, Wuhan, China), anti-TUBULIN (A12289, Abclonal, Wuhan, China), anti-H3 (AE092, Abclonal, Wuhan, China), anti-Flag (AE005, Abclonal, Wuhan, China), anti-GRP75 (A11256, Abclonal, Wuhan, China), anti-RFX1 (26859-1-AP, Proteintech, Wuhan, China), anti-pho- (AP1067, Abclonal, Wuhan, China), anti-PAK2 (A18093, Abclonal, Wuhan, China), anti-IP3R1 (A4436, Abclonal, Wuhan, China), anti-p-PAK2 (AP1158, Abclonal, Wuhan, China), anti-Ubi (A19686, Abclonal, Wuhan, China), anti-VDAC1 (A19707, Abclonal, Wuhan, China).

The following secondary antibodies were used: HRP-conjugated Goat anti-Rabbit IgG (AS014, Abclonal, Wuhan, China), HRP-conjugated Goat anti-Mouse IgG (AS003, Abclonal, Wuhan, China).

### 2.16. Immunofluorescence and Probe Staining

Immunofluorescence was performed as previously described [28]. Cells were fixed with 4% paraformaldehyde and permeabilized with 0.1% Triton X-100. To block non-specific antigen recognition, 5% BSA was used. After blocking for 30 min, the intended primary antibody was added at a dilution of 1:200 and incubated with the cells for 2 h at room temperature. Then, the anti-rabbit IgG-Cy3 conjugate (Servicebio, GB21303, Wuhan, China) and anti-mouse IgG-Alexa Fluor 488 (Servicebio, GB25301, Wuhan, China) were used to detect the primary antibody at a dilution of 1:200 for 1 h at room temperature. After that, cells were observed using confocal microscopy (Nikon, AX/AXR with NSPARC, Tokyo, Japan).

After dewaxing and rehydration, sections were incubated with 3% H_2_O_2_ to inactivate intrinsic peroxidase. To block non-specific antigen recognition, 5% BSA in 10% goat serum was used. After blocking for 1 h at room temperature, the primary antibody was used overnight at 4 °C. Then, anti-rabbit IgG-Cy3 conjugate (Servicebio, GB21303, Wuhan, China) and anti-mouse IgG-Alexa Fluor 488 (Servicebio, GB25301, Wuhan, China) were used to detect the primary antibody at a dilution of 1:200 for 1 h at room temperature. After that, sections were observed using confocal microscopy (Nikon, AX/AXR with NSPARC, Tokyo, Japan).

For probe staining, the intended probes (MitoTracker, ERTracker, Rhod-2 AM) were incubated with the cells at 37 °C in a cell culture incubator for 30 min at the concentrations recommended by the manufacturer. After washing with pre-warmed PBS twice, cells were observed using Nikon confocal microscopy.

The primary antibodies used were as follows: anti-Flag (AE005, Abclonal, Wuhan, China), anti-IP3R1 (A4436, Abclonal, Wuhan, China), anti-GRP75 (A11256, Abclonal, Wuhan, China), and anti-NeuN (A26951, Abclonal, Wuhan, China).

The commercial probes used were as follows: MitoTracker (Thermo Fisher Scientific, M7512, Waltham, MA, USA), ERTracker (Thermo Fisher Scientific, E12353, Waltham, MA, USA), Rhod-2 AM (S1062S, Beyotime, Shanghai, China).

### 2.17. Co-Immunoprecipitation

Co-immunoprecipitation was performed as previously described [29]. Following cell lysis with NP-40 lysis buffer and determination of protein concentration, 500 μg of total protein was incubated with anti-Flag magnetic beads (MCE, HY-K0207, Monmouth Junction, NJ, USA) overnight at 4 °C. Subsequently, the beads were washed three times with NP-40 lysis buffer and collected for subsequent experiments. To prepare for Western blot analysis, the beads were heated with 1× loading buffer for 10 min, and the resulting samples were subjected to Western blotting.

### 2.18. Luciferase Reporter Assay

Luciferase reporter assay was performed as previously described [30]. A DNA fragment containing the *Grp75* promoter sequence was inserted into the pGLuc-Basic vector (D2098, Beyotime, Shanghai, China) to generate a luciferase reporter construct harboring the *Grp75* promoter region (from −2000 bp to +200 bp). The reporter plasmid was co-transfected into cells with pcDNA3.1-Rfx1-Flag. After cell lysis, firefly and Renilla luciferase activities were measured using the Dual Luciferase Reporter Gene Assay Kit (RG027, Beyotime, Shanghai, China). The firefly/Renilla luminescence signal ratio was calculated for normalization.

### 2.19. Behavioural Tests

Open field test. Mice were placed individually in an open-field arena (40 × 40 × 40 cm) made of opaque Plexiglas and allowed to explore freely for 5 min under dim illumination. Total distance traveled, time spent in the centre zone, and rearing frequency were recorded automatically using a video tracking system (ANY-maze, Stoelting, Wood Dale, IL, USA). The test was used to assess general locomotor activity and anxiety-like behavior, and to exclude a locomotor explanation for any water-maze deficit. The arena was cleaned thoroughly with 75% ethanol between trials to eliminate residual odor cues.

Y-maze spontaneous alternation test. The Y-maze test was performed in a Y-shaped apparatus constructed from black polyvinyl chloride with three identical arms (each 28 cm long, 6 cm wide, and 18 cm high). The test was conducted under dim lighting in a quiet room. Each mouse was placed at the end of one arm and allowed to explore freely for 10 min. Arm entries were defined as the mouse entering an arm with all four paws. The sequence and number of arm entries were recorded using a video tracking system. Spontaneous alternation behavior was defined as successive entries into all three arms in overlapping triplet sets, excluding repeated sequences. The percentage of spontaneous alternation was calculated using the formula: Spontaneous alternation (%) = Number of alternations/(Total arm entries − 1) × 100%. The maze was cleaned thoroughly with 75% ethanol between trials to eliminate residual odor cues.

Morris water maze test. The Morris water maze test was performed in a circular pool (120 cm in diameter, 50 cm in height) filled with water maintained at 22 ± 1 °C and made opaque with non-toxic white paint. A transparent escape platform (10 cm in diameter) was placed 1 cm below the water surface in the center of the target quadrant. The test was conducted under dim lighting in a quiet room with distinct visual cues positioned around the pool. Mice were trained for 5 consecutive days, with four trials per day and an inter-trial interval of approximately 30 min. In each trial, the mouse was released from one of four pseudo-randomly assigned start positions and allowed to search for the hidden platform for a maximum of 60 s. If the mouse failed to locate the platform within this period, it was gently guided to the platform and allowed to remain there for 15 s. Escape latency, swimming speed, and distance traveled were recorded using a video tracking system. On day 6, a probe trial was conducted in which the platform was removed, and the mouse was allowed to swim freely for 60 s. The time spent in the target quadrant and the number of platform crossings were recorded to assess spatial memory retention. The pool was thoroughly cleaned and water temperature was maintained between trials to ensure consistent conditions.

All tests were performed by an investigator blinded to genotype and treatment.

### 2.20. Statistical Analysis

Statistical analysis was performed using GraphPad Prism 8.0.2. The normality of data distribution was assessed using the Shapiro–Wilk test. For comparisons between two groups, an unpaired two-tailed Student’s *t*-test was applied when data followed a normal distribution; otherwise, the Mann–Whitney U test was used. For multi-group comparisons, one-way ANOVA followed by Tukey’s post hoc test was performed when assumptions of normality and homogeneity of variances (tested by Levene’s test) were met; otherwise, the Kruskal–Wallis test with Dunn’s post hoc test was applied. Correlation analyses were conducted using Pearson’s correlation coefficient for normally distributed data and Spearman’s rank correlation coefficient for non-normally distributed data. All specific tests, exact *p*-values, and n values are reported in the figure legends. Group differences were considered significant at * *p* < 0.05, ** *p* < 0.01, *** *p* < 0.001, and **** *p* < 0.0001.

## 3. Results

### 3.1. Differential Nucleocytoplasmic DISC1 Distribution Correlates with Mitochondrial Ca^2+^ Overload in Diabetic Encephalopathy Neurons

**Figure 1:**
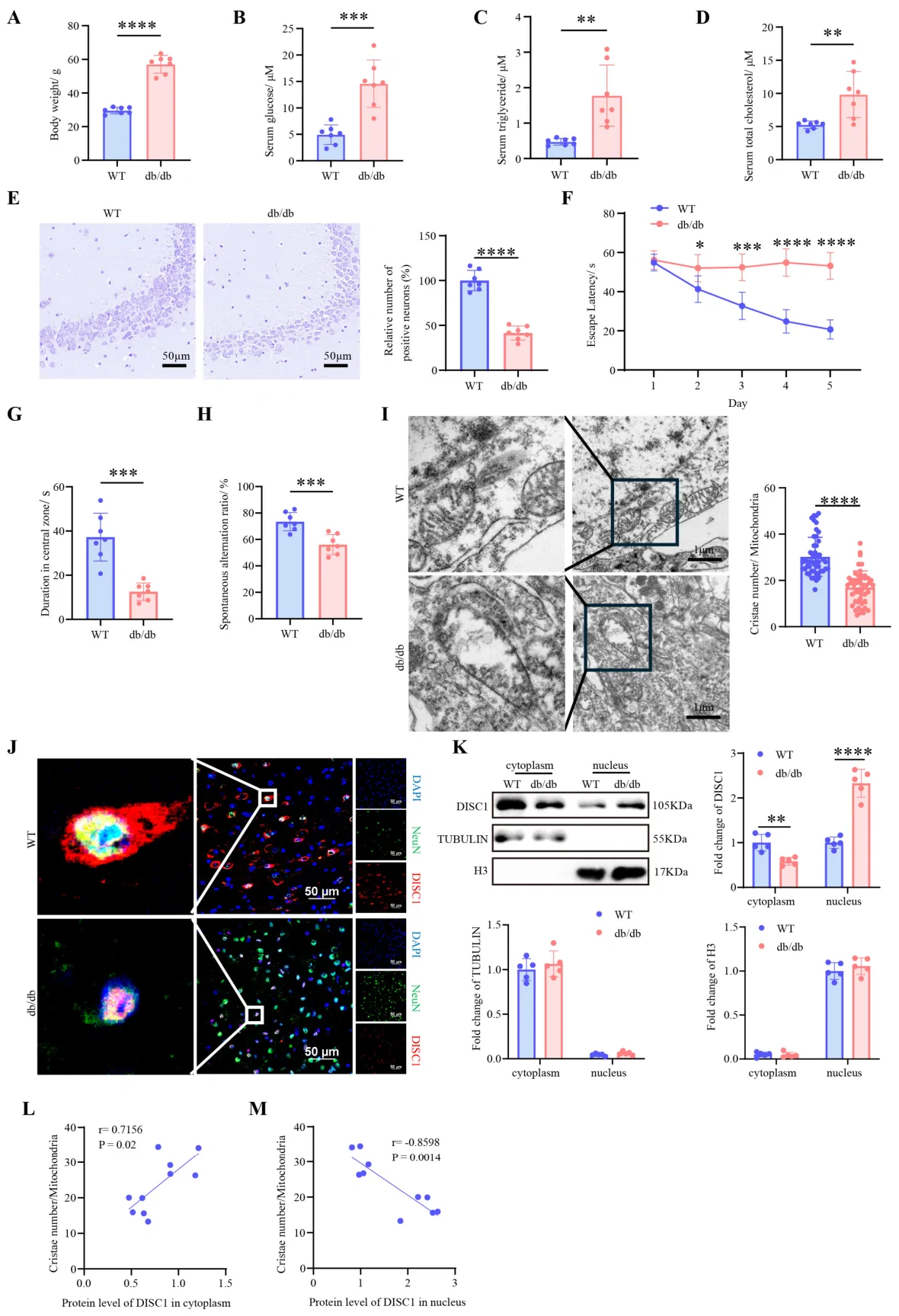
Differential nucleocytoplasmic DISC1 distribution correlates with mitochondrial Ca^2+^ overload in diabetic encephalopathy neurons. (**A**) Body weight of db/db mice and WT controls (*n* = 7 mice); (**B**–**D**) Serum triglyceride, total cholesterol, and glucose concentrations in db/db mice and WT controls (*n* = 7 mice); (**E**) Nissl staining of mouse brain sections in the db/db model (*n* = 7 mice); (**F**) Cognitive assessment by Morris water maze test (*n* = 7 mice); (**G**) Time spent in the center zone in the open field test; (**H**) Spontaneous alternation rate in the Y-maze test (*n* = 7 mice); (**I**) Electron microscopy visualization of mitochondrial ultrastructure in db/db mice versus WT mice (*n* = 49 mitochondria from 3 mice); (**J**) Immunofluorescence staining was performed to detect DISC1 in neurons of mouse brain sections; (**K**) Western blot analysis was performed to detect DISC1 protein levels in the nuclear and cytoplasmic fractions of mouse brain tissues (*n* = 5 mice); (**L**) Correlation analysis was performed between cytoplasmic DISC1 levels and the number of mitochondrial cristae in neurons (*n* = 10 mice); (**M**) Correlation analysis was performed between nuclear DISC1 levels and the number of mitochondrial cristae in neurons (*n* = 10 mice). Data are presented as mean ± SD. *p*-values were determined using an unpaired two-tailed Student’s *t*-test (**A**–**I**,**K**) and Pearson correlation (**L**,**M**). * *p* < 0.05, ** *p* < 0.01, *** *p* < 0.001, and **** *p* < 0.0001. The original western blot images can be found in Supplementary Materials.

To investigate the impact of a high-glucose environment on DISC1, the db/db mouse model of spontaneous diabetes was used. db/db mice exhibited significantly higher body weight, as well as elevated serum levels of glucose, triglycerides, and total cholesterol, compared with control mice (Figure 1A–D). Nissl staining in 12-week-old db/db mice revealed considerable neuronal damage (Figure 1E). Performance in the Morris water maze test indicated significantly slower learning rates in db/db mice, reflecting impaired cognitive function (Figure 1F). Moreover, in the open field test, db/db mice spent significantly less time in the center zone (Figure 1G). In the Y-maze test, the spontaneous alternation rate of db/db mice was significantly decreased (Figure 1H). Examination of brain tissues through electron microscopy showed a notable decrease in the number of mitochondrial cristae in neurons, indicating mitochondrial damage in the brains of db/db mice (Figure 1I). Immunofluorescence staining demonstrated a substantial increase in nuclear localization of DISC1 in neurons of db/db mice (Figure 1J). Subsequent nuclear and cytoplasmic fractionation revealed a significant reduction in cytoplasmic DISC1 and a notable increase in nuclear DISC1 in the brain tissues of db/db mice (Figure 1K). Correlation analysis revealed a significant positive correlation between nuclear DISC1 protein levels and the number of mitochondrial cristae in mouse brain tissues (Figure 1L), while the cytoplasmic DISC1 protein level exhibited a significant negative correlation with the number of mitochondrial cristae (Figure 1M). These findings suggest that the differential subcellular distribution of DISC1 in the nucleus and cytoplasm is linked to neuronal mitochondrial damage in diabetes.

**Figure 2:**
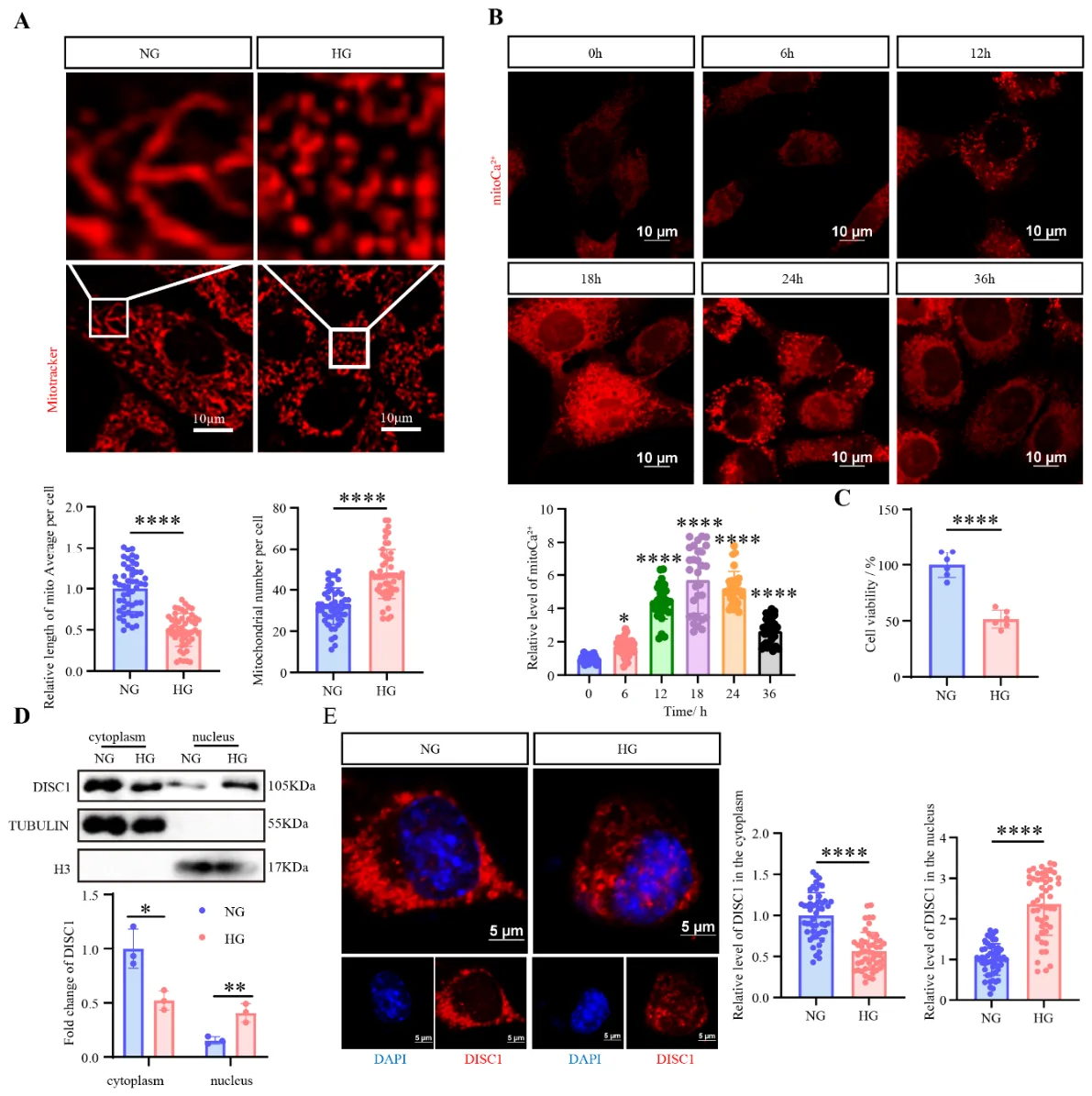
Differential nucleocytoplasmic DISC1 distribution correlates with mitochondrial Ca^2+^ overload in high-glucose-treated HT22 cells. (**A**) Mitochondrial morphology in HT22 cells treated with 50 mM glucose was assessed using MitoTracker (*n* = 50 cells from 5 independent cultures); (**B**) Mitochondrial Ca^2+^ levels in HT22 cells at different time points after high-glucose treatment were measured using a mitochondria-specific calcium indicator (*n* = 30 cells from 3 independent cultures); (**C**) Cell viability was evaluated using the CCK8 assay (*n* = 6 independent cultures); (**D**) Protein levels of cytoplasmic and nuclear DISC1 in HT22 cells under high-glucose treatment were determined (*n* = 3 independent cultures); (**E**) DISC1 localization in HT22 cells treated with high glucose was examined by immunofluorescence (*n* = 50 cells from 5 independent cultures). Data are presented as mean ± SD. *p*-values were determined using an unpaired two-tailed Student’s *t*-test (**A**,**C**–**E**) and one-way ANOVA followed by Tukey’s post hoc test (**B**). * *p* < 0.05, ** *p* < 0.01, and **** *p* < 0.0001.

In vitro, the culture medium was supplemented with 50 mM glucose to mimic hyperglycemic conditions associated with diabetes. Exposure to high glucose significantly increased mitochondrial fragmentation (Figure 2A). Subsequently, mitochondrial Ca^2+^ levels were assessed using the Rhod-2 AM probe. The results demonstrated a progressive increase in mitochondrial Ca^2+^ levels in HT22 cells following high-glucose treatment, peaking between 18–24 h and then decreasing (Figure 2B). Furthermore, cell viability was notably reduced after exposure to high glucose (Figure 2C). Corresponding with in vivo observations, the high-glucose environment led to a significant reduction in cytoplasmic DISC1 levels, while increasing nuclear DISC1 levels in HT22 cells (Figure 2D,E). However, in the microglial cell line BV2 and the astrocyte cell line C8-D1A, high-glucose treatment did not alter the nuclear localization of DISC1 (Figure S1A,B). Together, these findings suggest that the nuclear translocation of DISC1 and the decrease in cytoplasmic DISC1 may contribute to mitochondrial Ca^2+^ overload in neurons.

### 3.2. High-Glucose Drives DISC1-Nuclear Translocation to Induce Mitochondrial Ca^2+^ Overload via GRP75 Upregulation

**Figure 3:**
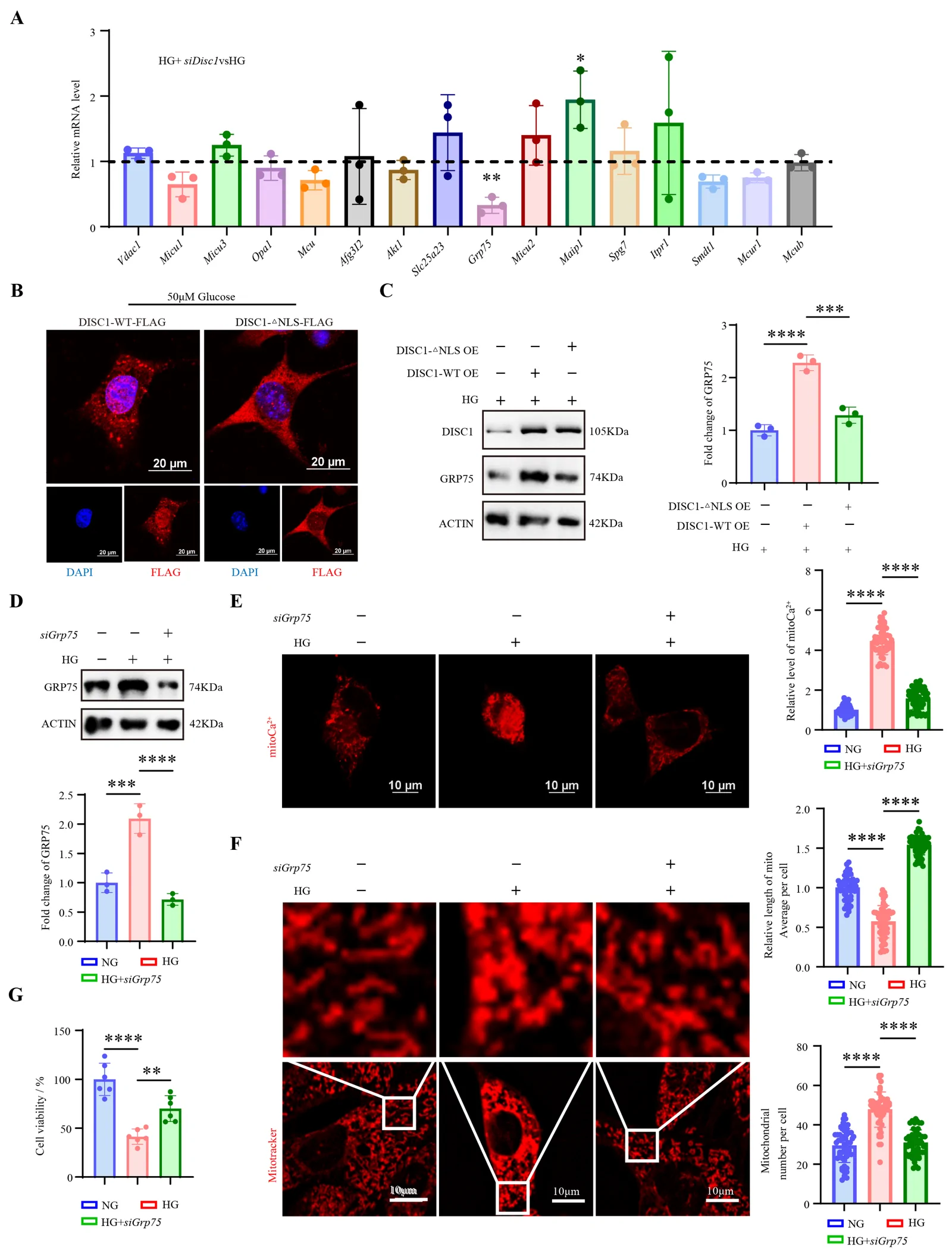
High glucose drives DISC1-nuclear translocation to induce mitochondrial Ca^2+^ overload via GRP75 upregulation. (**A**) The expression of genes related to “calcium import into the mitochondrion” from the GO database was examined in HT22 cells treated with high glucose following *Disc1* knockdown (*n* = 3 independent cultures); (**B**) Immunofluorescence was used to detect overexpressed wild-type DISC1 or NLS-deleted DISC1 in SH-SY5Y cells under high-glucose conditions (*n* = 3 independent cultures); (**C**) Western blot analysis was performed to detect GRP75 expression in SH-SY5Y cells overexpressing wild-type DISC1 or NLS-deleted DISC1 under high-glucose treatment (*n* = 3 independent cultures); (**D**) GRP75 expression was assessed in HT22 cells under high-glucose conditions following *Grp75* knockdown (*n* = 3 independent cultures); (**E**) Mitochondrial calcium levels were measured (*n* = 3); (**F**) Mitochondrial morphology was evaluated (*n* = 3 independent cultures); (**G**) Cell viability of HT22 cells was assessed (*n* = 3 independent cultures). Data are presented as mean ± SD. *p*-values were determined using an unpaired two-tailed Student’s *t*-test (**A**) and one-way ANOVA followed by Tukey’s post hoc test (**C**–**G**). * *p* < 0.05, ** *p* < 0.01, *** *p* < 0.001, and **** *p* < 0.0001.

To assess the role of nuclear DISC1 in regulating mitochondrial Ca^2+^ overload, *Disc1* was silenced using siRNA in HT22 cells, followed by exposure to high glucose. This was compared to HT22 cells treated solely with high glucose. Expression levels of genes associated with calcium import into the mitochondrion were assessed using qPCR, as identified in the Gene Ontology database (Figure 3A). The analysis revealed a significant reduction in *Grp75* mRNA levels following *Disc1* knockdown under high-glucose conditions (Figure 3A), suggesting a potential connection between nuclear DISC1 and *Grp75* expression in response to high-glucose stimulation.

Because the NLS of mouse DISC1 is unknown, a plasmid containing human DISC1 with a mutated NLS was generated to investigate the relationship between nuclear-localized DISC1 and GRP75 expression [23]. The NLS mutation abrogated the nuclear localization capability of DISC1 in SH-SY5Y cells (Figure 3B). Notably, overexpression of wild-type DISC1 led to increased GRP75 expression in SH-SY5Y cells, while overexpression of the NLS-deficient mutant DISC1 did not affect GRP75 expression (Figure 3C). This suggests that nuclear DISC1 activates GRP75 expression under high-glucose conditions. Subsequently, siRNA was used to knock down *Grp75* to mitigate high-glucose-induced damage in HT22 cells (Figure 3D). The results showed that *Grp75* knockdown significantly reduced mitochondrial Ca^2+^ levels, ameliorated mitochondrial fragmentation, and notably enhanced neuronal cell viability (Figure 3E–G). These results suggest that high glucose triggers DISC1 nuclear translocation, potentially leading to mitochondrial calcium overload through GRP75 upregulation. Collectively, these findings demonstrate that high-glucose-induced nuclear translocation of DISC1 enhances *Grp75* expression, contributing to mitochondrial Ca^2+^ overload and subsequent neuronal damage.

### 3.3. PAK2 Promotes DISC1 Nuclear Translocation via Inducing DISC1 Serine 285 Phosphorylation in High Glucose Conditions

**Figure 4:**
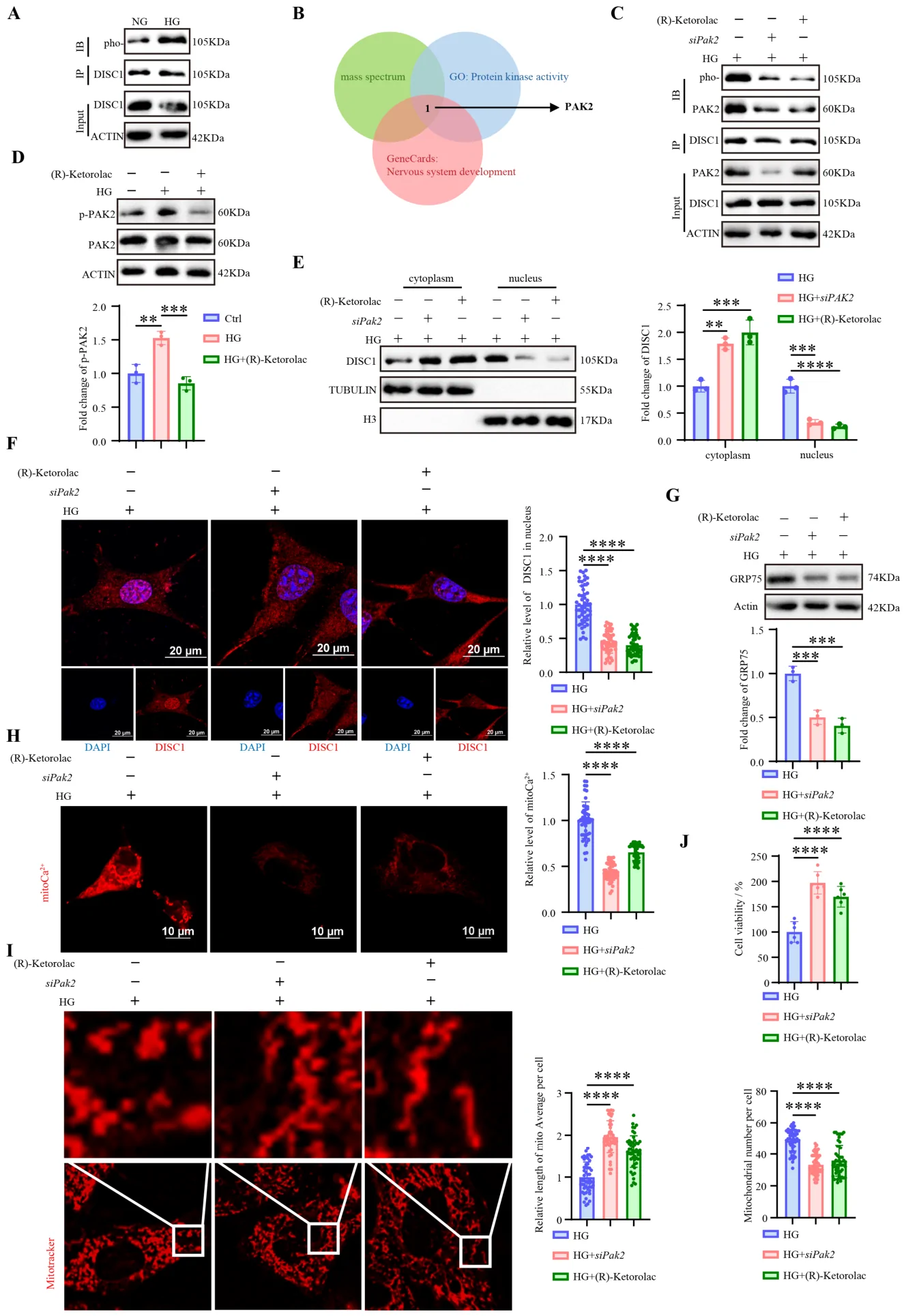
Pak2 promotes DISC1 phosphorylation and nuclear translocation in high-glucose conditions. (**A**) Co-immunoprecipitation followed by Western blot was performed to detect the phosphorylation level of DISC1; (**B**) After capturing DISC1 by co-immunoprecipitation, mass spectrometry was used to identify potential interacting proteins, which were then subjected to overlapping analysis with the “Protein kinase activity” gene set from the GO database and gene sets related to “Nervous system development” from the GeneCards database; (**C**) Following treatment with the CDC42/RAC1 inhibitor (R)-Ketorolac or knockdown of *Pak2*, co-immunoprecipitation followed by Western blot was performed to detect the phosphorylation level of DISC1 and the interaction between DISC1 and PAK2 in HT22 cells under high-glucose conditions; (**D**) The level of p-PAK2 was detected by Western blot (*n* = 3 independent cultures); (**E**) The levels of cytoplasmic and nuclear DISC1 were detected by Western blot (*n* = 3 independent cultures); (**F**) DISC1 localization was examined by immunofluorescence (*n* = 50 cells from 5 independent cultures); (**G**) The level of GRP75 was detected by Western blot (*n* = 3 independent cultures); (**H**) Mitochondrial calcium levels were measured (*n* = 50 cells from 5 independent cultures); (**I**) Mitochondrial morphology was assessed (*n* = 50 cells from 5 independent cultures); (**J**) Cell viability of HT22 cells was evaluated (*n* = 6 independent cultures). Data are presented as mean ± SD. *p*-values were determined using one-way ANOVA followed by Tukey’s post hoc test (**D**–**J**). ** *p* < 0.01, *** *p* < 0.001, and **** *p* < 0.0001.

Porteous et al. proposed that phosphorylation of DISC1 may regulate its nucleocytoplasmic shuttling [23]. To investigate this, the phosphorylation level of DISC1 was examined via Western blot following co-immunoprecipitation (Figure 4A). It was observed that high glucose treatment significantly increased the phosphorylation of DISC1 (Figure 4A). Mass spectrometry identified endogenous phosphorylation sites at residues S284 and S285 of DISC1 in a high-glucose environment (Figure S1C).

To determine the kinase responsible for the phosphorylation of DISC1 at serine residues, co-immunoprecipitation was performed to capture DISC1-interacting proteins, followed by mass spectrometry analysis. The resulting protein list was then compared with the gene set associated with “Protein kinase activity” from the Gene Ontology database and with gene sets related to “Nervous system development” from the GeneCards database (Figure 4B). Among the kinases identified in the DISC1 interactome (JAK1, PKM, PGK1, PAK2), only PAK2 is a serine/threonine kinase; JAK1 is a tyrosine kinase and PKM/PGK1 are glycolytic enzymes, indicating PAK2 as the sole candidate for direct DISC1 phosphorylation (Figure 4B). Consequently, it was hypothesized that the serine/threonine kinase Pak2 could mediate DISC1 phosphorylation. This hypothesis was supported by the confirmation of the interaction between DISC1 and PAK2 through co-immunoprecipitation and GST pull-down (Figure 4C and Figure S1F). Importantly, siRNA-mediated knockdown of *Pak2* resulted in a significant reduction in the phosphorylation level of DISC1 (Figure 4C).

PAK1 has been shown to enhance its kinase activity through autophosphorylation [31]. Consistently, Western blotting demonstrated a significant increase in PAK2 phosphorylation following high-glucose treatment (Figure 4C). Research indicates that diabetes triggers PAK2 autophosphorylation via the CDC42/RAC1 signaling pathway [32]. Treatment with the CDC42/RAC1 inhibitor (R)-Ketorolac effectively suppressed the high-glucose-induced phosphorylation of PAK2 and DISC1 (Figure 4C). Importantly, both *Pak2* knockdown and (R)-Ketorolac treatment markedly suppressed the high-glucose-induced increase in DISC1 nuclear localization (Figure 4E,F). Furthermore, *Pak2* knockdown and (R)-Ketorolac mitigated the high-glucose-induced upregulation of GRP75 expression, decreased mitochondrial Ca^2+^ levels, ameliorated mitochondrial fragmentation, and improved neuronal viability following high-glucose exposure (Figure 4G–J).

**Figure 5:**
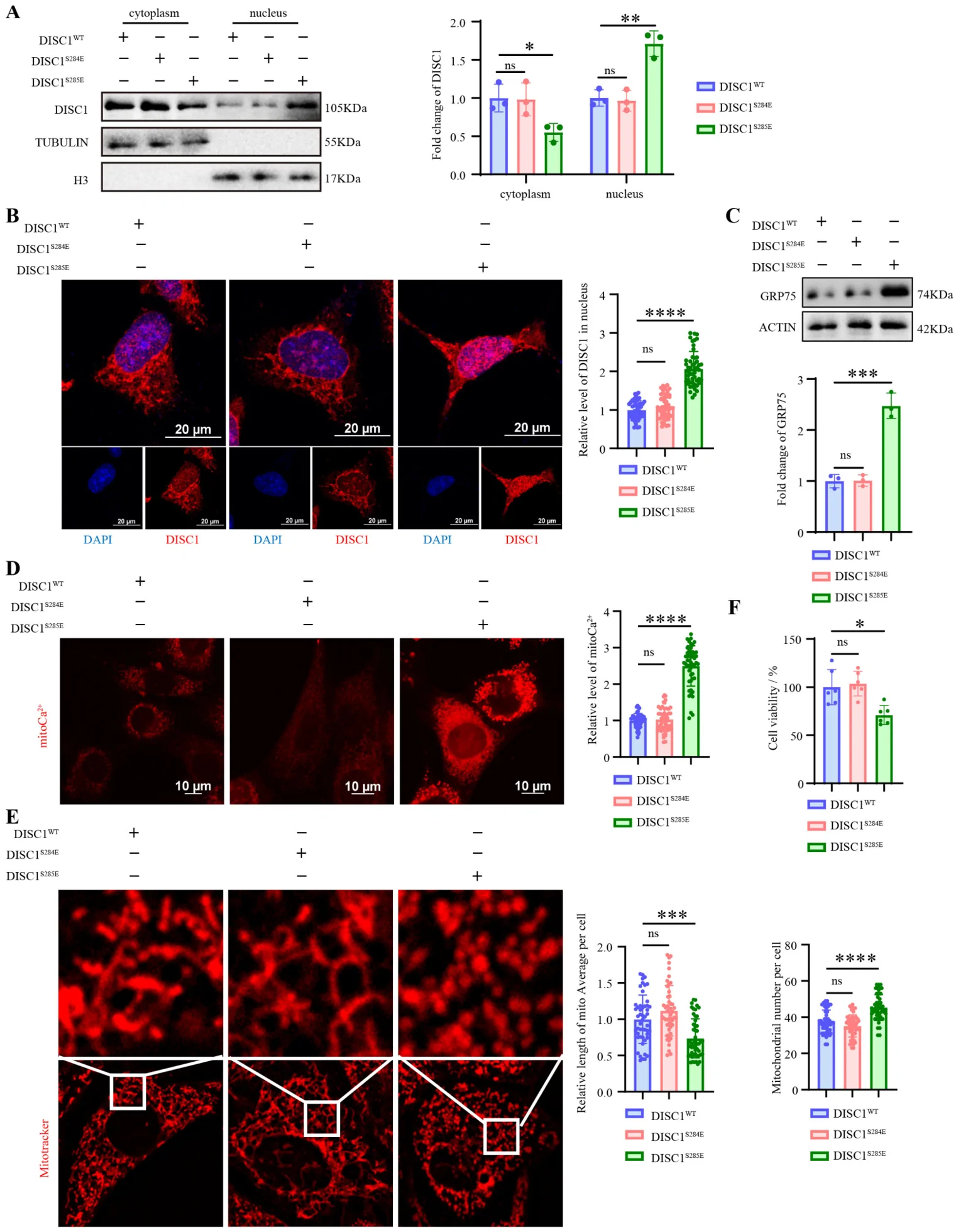
Phosphorylation of DISC1 at serine 285 drives its nuclear translocation. (**A**) Serine 284 or serine 285 of wild-type DISC1 was mutated to glutamic acid to generate DISC1^S284E^ and DISC1^S285E^. After overexpression of the three DISC1 constructs, Western blot was performed to detect cytoplasmic and nuclear DISC1 levels in SH-SY5Y cells (*n* = 3 independent cultures); (**B**) DISC1 localization was examined by immunofluorescence (*n* = 50 cells from 5 independent cultures); (**C**) GRP75 levels were detected by Western blot (*n* = 3 independent cultures); (**D**) Mitochondrial calcium levels were measured (*n* = 50 cells from 5 independent cultures); (**E**) Mitochondrial morphology was assessed (*n* = 50 cells from 5 independent cultures); (**F**) Cell viability of HT22 cells was evaluated (*n* = 6 independent cultures). Data are presented as mean ± SD. *p*-values were determined using a one-way ANOVA followed by Tukey’s post hoc test (**A**–**F**). * *p* < 0.05, ** *p* < 0.01, *** *p* < 0.001, and **** *p* < 0.0001.

Then, plasmids encoding wild-type DISC1 (DISC1^WT^) and mutants mimicking constitutive phosphorylation at serine 284 (DISC1S^284E^) or serine 285 (DISC1^S285E^) were constructed to identify phosphorylated residues of DISC1. Overexpression of DISC1^S285E^ in SH-SY5Y cells led to significantly higher nuclear localization compared to DISC1^WT^ or DISC1^S284E^ (Figure 5A,B), accompanied by elevated GRP75 protein expression (Figure 5C). Additionally, DISC1^S285E^ overexpression resulted in elevated mitochondrial Ca^2+^ levels, mitochondrial fragmentation, and reduced neuronal cell viability (Figure 5D–F).

To further investigate the functional role of DISC1 phosphorylation at serine 285, we generated a phospho-dead mutant by substituting S285 with alanine (DISC1^S285A^). Following DISC1 knockdown, SH-SY5Y cells were rescued with either DISC1^WT^ or DISC1^S285A^ under high-glucose conditions. Subcellular localization analysis revealed that DISC1^S285A^ effectively prevented the high-glucose-induced nuclear translocation of DISC1 compared with DISC1^WT^ (Figure S2A,B). Moreover, DISC1^S285A^ overexpression led to reduced GRP75 protein levels relative to DISC1^WT^ rescue (Figure S2C). Furthermore, compared with DISC1^WT^ rescue, DISC1^S285A^ rescue significantly reduced mitochondrial Ca^2+^ levels, improved mitochondrial homeostasis, and enhanced neuronal viability under high-glucose conditions (Figure S2D–F).

Collectively, these findings highlight serine 285 phosphorylation as a critical determinant of DISC1 nuclear translocation and GRP75-mediated mitochondrial dysfunction. Furthermore, in a high-glucose environment, Pak2 activation through CDC42/RAC1 promotes DISC1 phosphorylation and nuclear translocation, leading to GRP75-mediated mitochondrial Ca^2+^ overload.

### 3.4. Nuclear DISC1 Activates Grp75 Transcription as a Coactivator for RFX1

**Figure 6:**
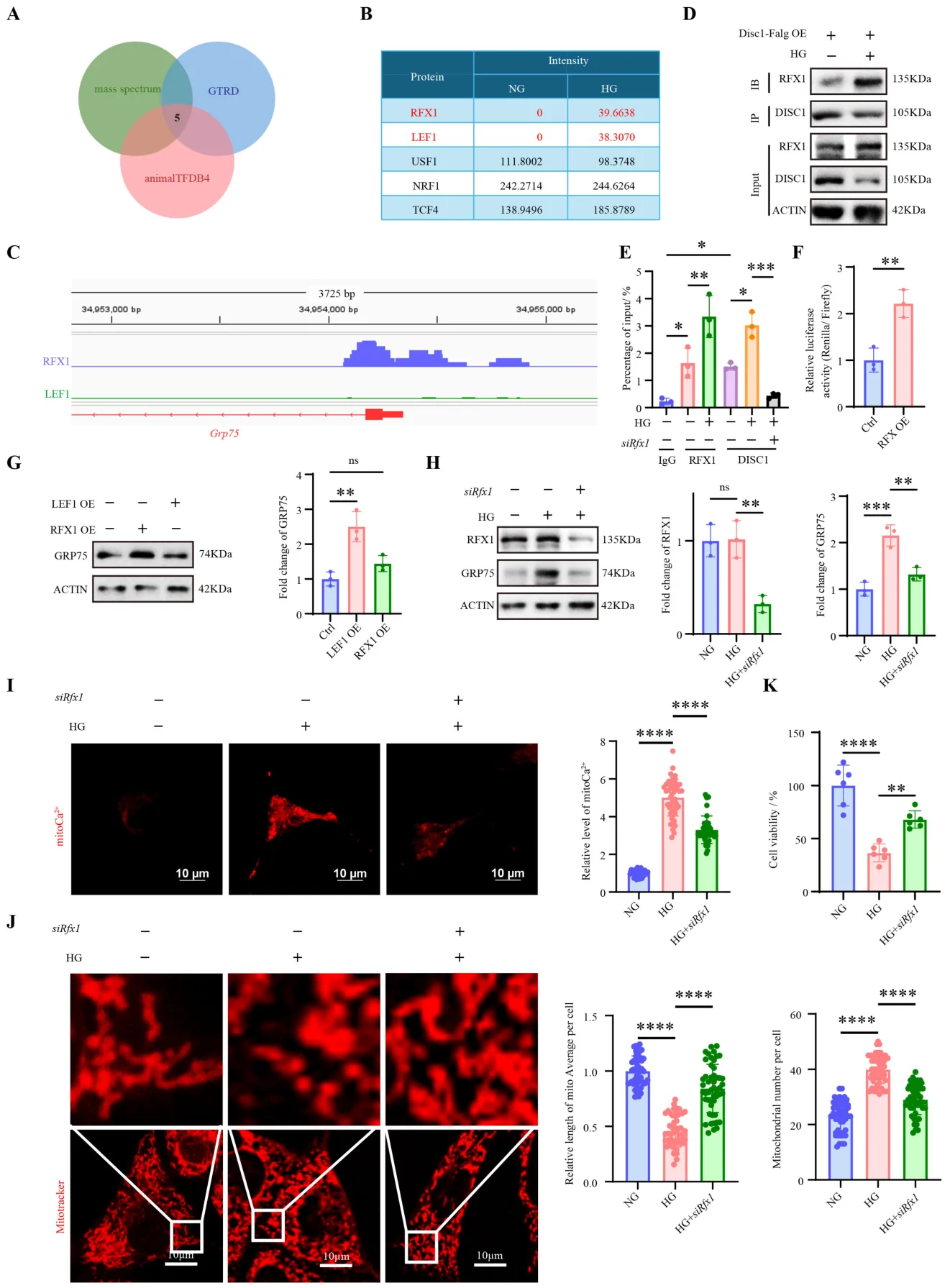
Nuclear DISC1 activates *Grp75* transcription through interaction with transcription factor RFX1. (**A**) Mass spectrometry identification of nuclear DISC1-interacting proteins combined with bioinformatic prediction of *Grp75* transcription factors using GTRD and AnimalTFDB4 databases, presented as a Venn diagram of three datasets; (**B**) Five candidate proteins from the overlapping segment of panel A; (**C**) Enrichment profiles of RFX1 and LEF1 at the *Grp75* promoter region; (**D**) Co-immunoprecipitation validation of DISC1-RFX1 interaction; (**E**) chromatin immunoprecipitation (ChIP)-qPCR quantification of RFX1 or DISC1 enrichment at the *Grp*75 promoter (*n* = 3 independent cultures); (**F**) Luciferase reporter assays were performed to evaluate the regulatory effect of RFX1 on genes driven by the *Grp75* promoter (*n* = 3 independent cultures); (**G**) Western blot analysis of GRP75 protein levels following *Rfx1* or *Lef1* overexpression (*n* = 3 independent cultures); (**H**) Western blot detection of RFX1 and GRP75 expression in high-glucose-treated HT22 cells after *Rfx1* knockdown (*n* = 3 independent cultures); (**I**) Mitochondrial morphology was assessed (*n* = 50); (**J**) Mitochondrial Ca^2+^ levels measured with a fluorescent probe (*n* = 50 cells from 5 independent cultures); (**K**) Cell viability assessed by CCK-8 assay (*n* = 6 independent cultures). *p*-values were determined using an unpaired two-tailed Student’s *t*-test (**E**,**F**) and one-way ANOVA followed by Tukey’s post hoc test (**G**–**K**). * *p* < 0.05, ** *p* < 0.01, *** *p* < 0.001, and **** *p* < 0.0001.

To elucidate the molecular mechanism through which nuclear DISC1 modulates *Grp75* expression, immunoprecipitation was performed to discover DISC1-interacting proteins, followed by mass spectrometry analysis. Subsequently, potential transcription factors associated with the regulation of *Grp75* transcription were predicted utilizing the GTRD and AnimalTFDB4 databases (Figure 6A). Of note, from the mass spectrometry data and predicted transcription factors, five candidates were identified (Figure 6A). Among them, interactions of DISC1 with RFX1 and LEF1 were notably enhanced under high-glucose conditions (Figure 6B). Database searches in Cistrome DB revealed a significant enrichment of RFX1 at the *Grp75* promoter region (Figure 6C). Immunoprecipitation with Western blot analysis confirmed the interaction between DISC1 and RFX1 under high-glucose conditions (Figure 6D). Chromatin immunoprecipitation (ChIP)-qPCR analysis validated the increase in RFX1 enrichment at the *Grp75* promoter under high-glucose conditions in HT22 cells (Figure 6E). Furthermore, high glucose significantly increased DISC1 enrichment at the *Grp75* promoter, whereas *Rfx1* knockdown markedly reduced DISC1 enrichment at this region (Figure 6E). Moreover, overexpression of DISC1^S285E^ significantly increased RFX1 enrichment at the *GRP75* promoter compared with overexpression of DISC1^WT^ or DISC1^S284E^ in SH-SY5Y cells (Figure S1D). Conversely, following *DISC1* knockdown, rescue with DISC1^S285A^ markedly reduced RFX1 enrichment at the *GRP75* promoter relative to rescue with DISC1^WT^ in SH-SY5Y cells (Figure S1E). In vitro kinase assays further demonstrated that PAK2 mediated the phosphorylation of wild-type DISC1, rather than that of the S285A mutant (Figure S1G). Consistently, luciferase reporter assays further demonstrated that RFX1 directly activates transcription from the *Grp75* promoter (Figure 6F). *Rfx1* overexpression, but not *Lef1*, increased GRP75 protein levels (Figure 6G), while *Rfx1* knockdown using siRNA attenuated the high-glucose-induced elevation in GRP75 protein levels (Figure 6H). These results suggest that elevated glucose levels promote the association of DISC1 with RFX1, enhancing RFX1 binding at the *Grp75* promoter and increasing GRP75 expression. Consistent with these findings, reducing RFX1 mitigated mitochondrial Ca^2+^ overload, ameliorated mitochondrial fragmentation, and improved cell viability (Figure 6I–K).

We further validated this mechanism in primary neurons. High-glucose treatment also promoted the nuclear localization of DISC1, while knockdown of Pak2 reduced DISC1 nuclear localization in primary neurons (Figure S3A). In parallel, high-glucose treatment increased DISC1 phosphorylation levels and the interaction between DISC1 and RFX1, whereas *Pak2* knockdown decreased both DISC1 phosphorylation and the DISC1–RFX1 interaction in primary neurons (Figure S3B). Finally, high-glucose treatment promoted *Grp75* mRNA expression and mitochondrial Ca^2+^ overload, while knockdown of either *Pak2* or *Rfx1* significantly reduced *Grp75* mRNA levels and mitochondrial Ca^2+^ overload (Figure S3C,D).

**Figure 7:**
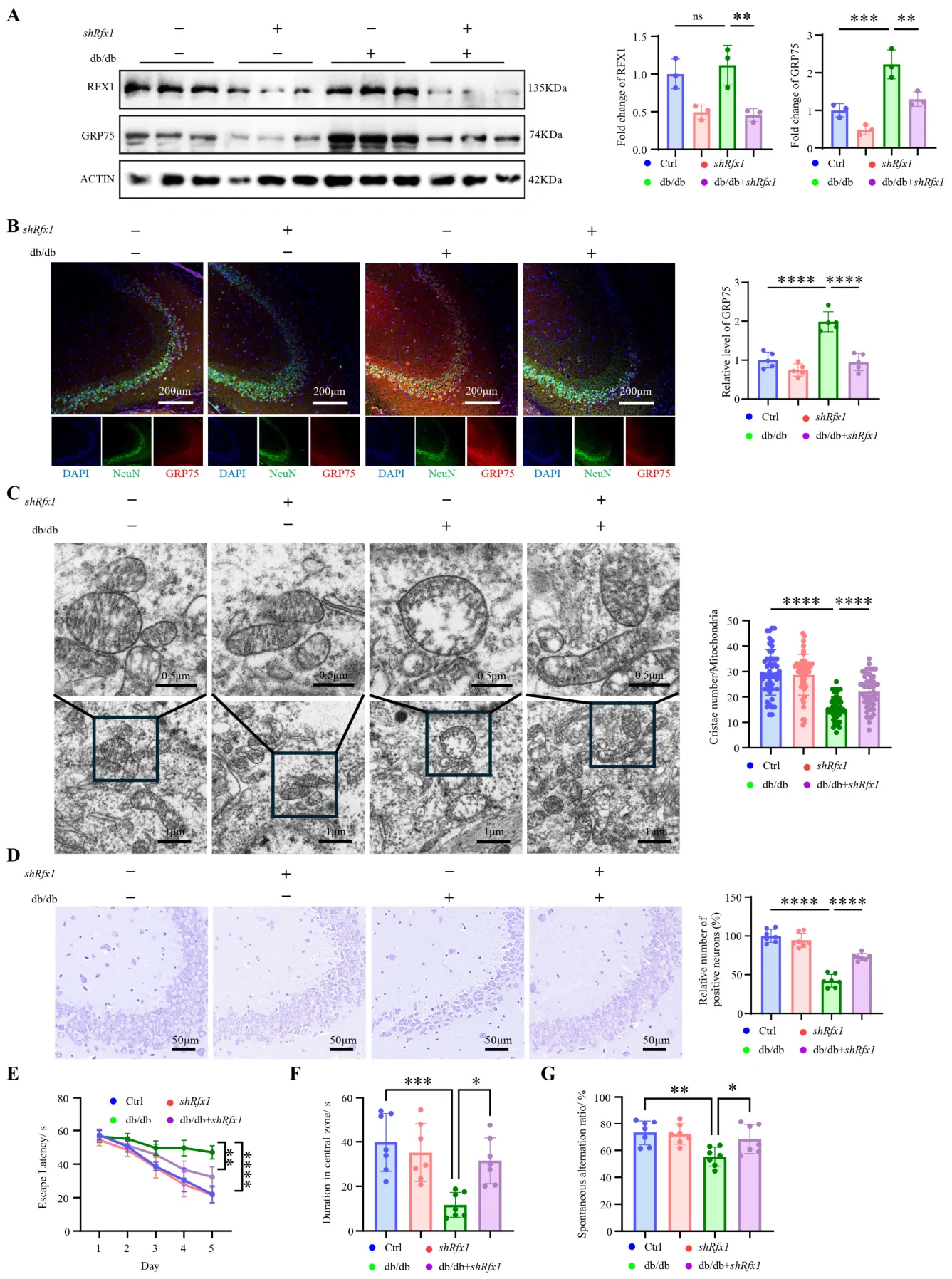
Knockdown of *Rfx1* ameliorates diabetic encephalopathy. (**A**) Western blot analysis of RFX1 and GRP75 protein levels in db/db mice following neuron-specific *Rfx1* knockdown via AAV9-hSyn-shRNA (*n* = 3 mice); (**B**) Immunofluorescence assessment of GRP75 in neurons (*n* = 5 mice); (**C**) Electron microscopy visualization of mitochondrial ultrastructure in neurons (*n* = 50 mitochondria from 3 mice); (**D**) Nissl staining of mouse brain sections (*n* = 5 mice); (**E**) Cognitive function evaluation by Morris water maze test (*n* = 5 mice); (**F**) Time spent in the center zone in the open field test; (**G**) Spontaneous alternation rate in the Y-maze test (*n* = 7 mice). Data are presented as mean ± SD. *p*-values were determined using one-way ANOVA followed by Tukey’s post hoc test (**A**–**G**). * *p* < 0.05, ** *p* < 0.01, *** *p* < 0.001, and **** *p* < 0.0001.

At the animal level, we conducted in vivo testing of the aforementioned findings. We utilized AAV9-delivered shRNA under the control of the hSyn promoter to specifically reduce *Rfx1* levels in cerebral neurons. The knockdown of *Rfx1* significantly mitigated the diabetes-induced elevation in GRP75 protein levels (Figure 7A,B), restored mitochondrial cristae abundance, and alleviated diabetes-induced neuronal damage and cognitive deficits (Figure 7C–G). The findings support the concept that increased nuclear localization of DISC1 triggers upregulation of the *Grp75* gene through its interaction with RFX1. This, in turn, results in GRP75-mediated mitochondrial Ca^2+^ overload, playing a role in the development of diabetic encephalopathy.

### 3.5. High-Glucose Downregulates Cytosolic DISC1, Inducing Mitochondrial Ca^2+^ Overload

To determine whether decreased DISC1 levels are an important mechanism of mitochondrial Ca^2+^ overload in DE neurons, we knocked down *Disc1* using siRNA (Figure S4A). This knockdown led to a significant increase in ER-mitochondria colocalization (Figure S4B), along with mitochondrial Ca^2+^ overload (Figure S4C), mitochondrial fragmentation (Figure S4B), and reduced HT22 cell viability (Figure S4D). Furthermore, transfection of NLS-mutated *DISC1* plasmid into high-glucose-treated SH-SY5Y cells resulted in a partial reversal of high-glucose-induced mitochondrial Ca^2+^ overload, fragmentation, and cell damage (Figure S5A–C), suggesting a contribution of cytoplasmic DISC1 loss to these pathological changes. Moreover, using a luciferase reporter assay, we found that overexpression of wild-type DISC1 significantly enhanced the transcriptional activity of RFX1 at the *GRP75* promoter, whereas overexpression of the NLS-mutated DISC1 failed to do so (Figure S5D).

**Figure 8:**
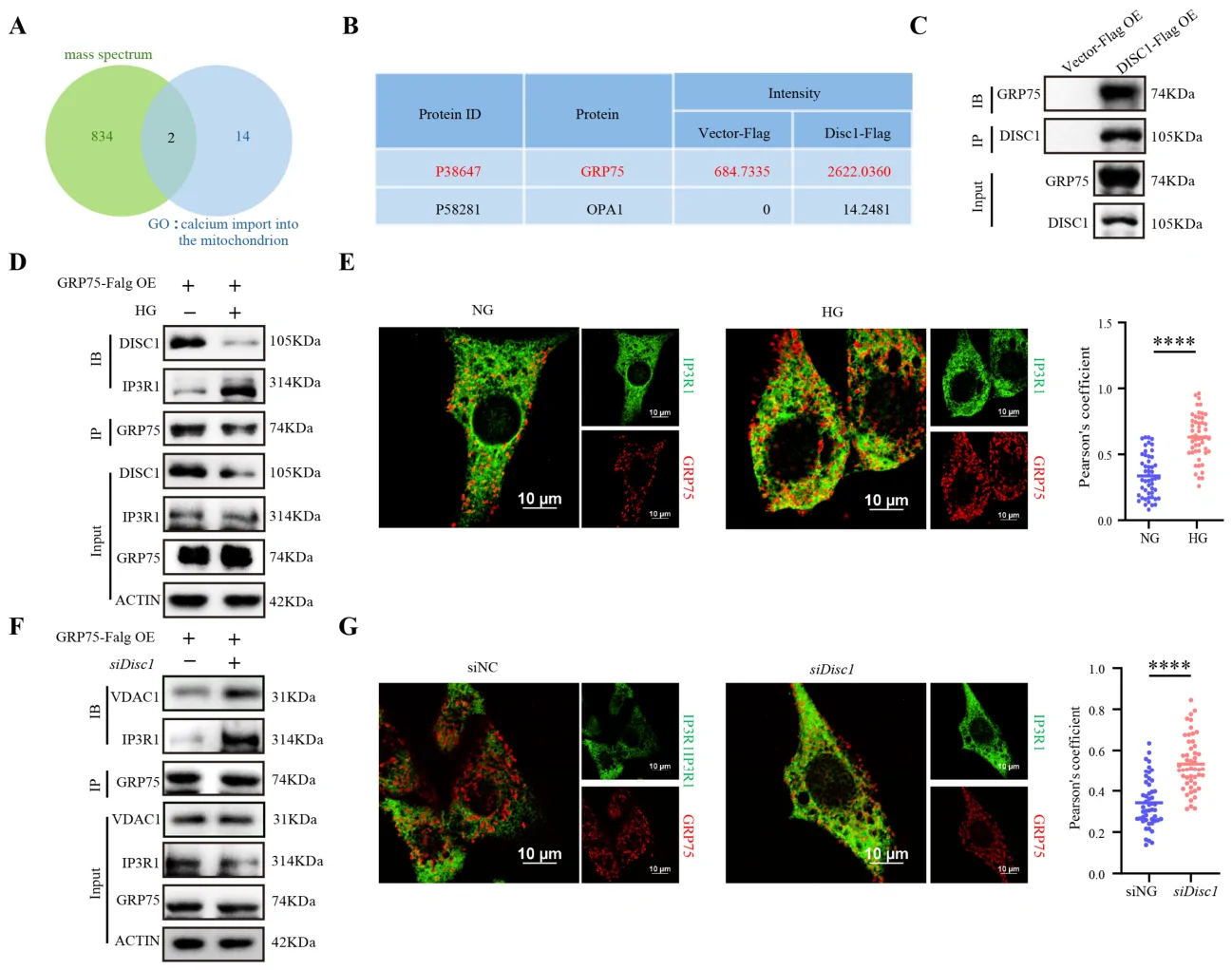
Cytoplasmic DISC1 impedes GRP75/IP3R1/VDAC1 complex-mediated mitochondrial calcium overload by interacting with GRP75. (**A**) Co-immunoprecipitation followed by mass spectrometry to identify DISC1-interacting proteins, with cross-analysis against the “calcium import into the mitochondrion” gene set from the Gene Ontology (GO) database presented as a Venn diagram; (**B**) Two candidate genes from the intersection; (**C**) Co-immunoprecipitation assays examining interactions between DISC1 and GRP75; (**D**) Co-immunoprecipitation analysis of GRP75 interactions with DISC1, IP3R1 and GRP75 under high-glucose conditions; (**E**) Immunofluorescence colocalization of GRP75 and IP3R1 (*n* = 50 cells from 5 independent cultures); (**F**) Co-immunoprecipitation detection of GRP75-IP3R1 interaction and GRP75-VDAC1 interaction following *Disc1* knockdown by siRNA; (**G**) Immunofluorescence assessment of GRP75 and IP3R1 colocalization after DISC1 knockdown (*n* = 50 cells from 5 independent cultures). Data are presented as mean ± SD. *p*-values were determined using an unpaired two-tailed Student’s *t*-test (**E**,**G**). **** *p* < 0.0001.

To elucidate the molecular mechanism underlying the contribution of reduced cytoplasmic DISC1 to mitochondrial Ca^2+^ overload, DISC1 was immunoprecipitated and subjected to mass spectrometry analysis to identify potential interacting proteins. The proteins identified were cross-analyzed with the “calcium import into the mitochondrion” gene set from the Gene Ontology database (Figure 8A), revealing four proteins, with GRP75 showing the highest abundance (Figure 8B). Subsequent co-immunoprecipitation and Western blot analyses confirmed the interaction between DISC1 and GRP75 (Figure 8C). Notably, under high-glucose conditions, the interaction between GRP75 and DISC1 was enhanced (Figure 8D). Given that the GRP75/IP3R1/VDAC1 complex facilitates the transfer of Ca^2+^ from the ER to mitochondria, we propose that the DISC1-GRP75 interaction modulates mitochondrial Ca^2+^ transport by influencing this complex. Interestingly, the GRP75-IP3R1 interaction was significantly increased in a high-glucose environment (Figure 8D,E). Knockdown of *Disc1* using siRNA also notably enhanced the interaction between GRP75 and IP3R1, as well as the interaction between GRP75 and VDAC1 (Figure 8F,G). These findings suggest that cytoplasmic DISC1 binds GRP75 and limits the assembly of the GRP75-IP3R1 module of the GRP75/IP3R1/VDAC1 complex, thereby preventing mitochondrial Ca^2+^ overload. Downregulation of cytoplasmic DISC1 induced by high glucose relieves this inhibition, leading to neuronal Ca^2+^ overload.

## 4. Discussion

As one of the most serious complications of diabetes, cognitive impairment affects millions of patients with diabetes. Investigating the pathogenesis of DE can provide a crucial theoretical foundation for identifying novel therapeutic targets. In this study, we found that a high-glucose environment drives neuronal mitochondrial Ca^2+^ overload via a novel nucleocytoplasmic “dual-effect” of DISC1. On the one hand, high glucose activates Pak2-mediated DISC1 phosphorylation to induce DISC1 nuclear translocation. As a coactivator for the transcription factor Rfx1, nuclear DISC1 activates the expression of *Grp75*, which facilitates Ca^2+^ transfer from the endoplasmic reticulum to the mitochondria by forming a GRP75/IP3R1/VDAC1 complex. On the other hand, cytoplasmic DISC1 can interact with Grp75, thereby inhibiting the formation of the GRP75/IP3R1/VDAC1 complex. However, the high-glucose environment promotes the reduction of cytoplasmic DISC1 by promoting its nuclear translocation.

A particularly compelling observation from this study is the divergence in DISC1 distribution: high-glucose treatment leads to an increase in nuclear DISC1 and a corresponding decrease in cytoplasmic DISC1. The presence of DISC1 in the nucleus was observed over two decades ago [23], yet the regulatory mechanisms underlying its nuclear translocation remained unclear. One possible reason is that, in most cases, DISC1 predominantly resides in the cytoplasm and has not been closely examined in the nuclear context. In 2005, Porteous et al. hypothesized that nuclear localization of DISC1 might depend on its phosphorylation status [23]. Our analysis of potential phosphorylation sites on DISC1 using the PhosphoSite database revealed numerous putative phosphorylation sites, many of which are located near its NLS and putative nuclear export sequences. Therefore, we conducted further experimental validation. Fortunately, we discovered that CDC42/RAC1 regulates DISC1 phosphorylation and nuclear localization through Pak2. We also note that (R)-Ketorolac acts upstream of PAK2 at the level of CDC42/RAC1 and may affect other CDC42/RAC1 effectors, and that it has activities independent of this pathway; the inhibitor and the PAK2 knockdown experiments are therefore mutually corroborating rather than individually sufficient. Pak2 knockdown reduced, but did not abolish, the high-glucose-induced increase in DISC1 phosphorylation, indicating that additional, unidentified kinases also contribute. The interactome screen that nominated PAK2 is subject to the abundance and affinity biases inherent in affinity-capture mass spectrometry, and its failure to detect other kinases is not evidence of their absence. This finding enhances our understanding of DISC1 function and identifies the CDC42/RAC1-PAK2-DISC1 axis as a candidate for future pharmacological investigation in DE.

Since DISC1 lacks a DNA-binding domain, it cannot act as a direct transcription factor. Instead, DISC1 may function as a transcriptional coactivator, regulating gene transcription through interactions with transcription factors [24,33]. In psychiatric disorder models, the interaction between DISC1 and ATF4 has been shown to cause synaptic dysregulation [24]. Moreover, Tsai et al. demonstrated that DISC1 forms a transcriptional repressor complex with ATF4 to regulate neuronal gene expression [33]. Our study reveals that DISC1 can also act as a coactivator of transcription factor Rfx1 to promote Grp75 transcription in neurons, further highlighting its critical role as a transcriptional co-regulator. Further identification of the precise promoter sequences bound by RFX1 will be necessary to fully define the regulatory elements involved. Moreover, whether DISC1-RFX1 has additional downstream target genes and whether DISC1 also acts through components of the mitochondrial Ca^2+^ uptake machinery other than GRP75 remain to be systematically investigated.

The molecular mechanism by which Ser285 phosphorylation facilitates DISC1 nuclear translocation remains to be fully defined. One possibility is that phosphorylation at Ser285 induces a conformational change that exposes an otherwise buried nuclear localization signal within the N-terminal head domain of DISC1, thereby promoting recognition by nuclear import machinery. Alternatively, Ser285 phosphorylation may mask a nuclear export signal, reducing DISC1 export from the nucleus and favoring its nuclear retention. Structural studies will be required to distinguish between these possibilities and to define the precise conformational consequences of Ser285 phosphorylation. These questions represent important directions for future investigation.

As the major intracellular Ca^2+^ store, the concentration of Ca^2+^ in the ER is much higher than that in the cytosol [34,35]. Hence, the outflow of Ca^2+^ from the ER needs precise regulation to prevent excessive Ca^2+^ release [36]. Mitochondria are important buffering organelles for ER Ca^2+^ [37]. However, excessive Ca^2+^ inflow ultimately leads to mitochondrial Ca^2+^ overload and may cause mitochondrial dysfunction [38]. Here, we provide evidence that DISC1 binds Grp75 and limits the assembly of the GRP75-IP3R1 module of the GRP75/IP3R1/VDAC1 complex, thereby opposing mitochondrial Ca^2+^ overload. Park et al. (2017) demonstrated that DISC1 binds to IP3R1 to block GRP75/IP3R1/VDAC1 complex-mediated mitochondrial Ca^2+^ overload in neurons [22]. In the present study, we found that under high-glucose conditions, in addition to its direct cytoplasmic “gatekeeper” function, DISC1 translocates to the nucleus in a PAK2-dependent manner and promotes GRP75 transcription via RFX1. This nuclear pathway is distinct from the previously described cytoplasmic scaffolding role: while the latter physically constrains the GRP75/IP3R1/VDAC1 complex at the ER–mitochondria interface, the former regulates the expression of a key component of this complex. Collectively, loss of cytoplasmic DISC1 and its concurrent nuclear accumulation cooperate to upregulate GRP75 and trigger mitochondrial Ca^2+^ overload. From a translational perspective, these findings refine our understanding of DISC1’s dual roles in different subcellular compartments and suggest that therapeutic strategies aimed solely at preserving cytoplasmic DISC1 may be insufficient. A more effective approach may require simultaneous restoration of cytoplasmic DISC1 function and prevention of its nuclear translocation. Furthermore, how DISC1 blocks the assembly of the GRP75/IP3R1/VDAC1 complex still requires further investigation.

From a translational perspective, the PAK2-DISC1-RFX1-GRP75 axis may offer several potential therapeutic opportunities for diabetic encephalopathy. First, inhibition of PAK2 or prevention of DISC1 Ser285 phosphorylation could limit DISC1 nuclear translocation and thereby reduce GRP75 expression, potentially restoring mitochondrial Ca^2+^ homeostasis. Second, disrupting the DISC1–RFX1 interaction may provide a more selective approach to block GRP75 transcription without affecting the cytoplasmic gatekeeping function of DISC1. Third, normalizing GRP75 expression or inhibiting the GRP75/IP3R1/VDAC1 complex may directly attenuate pathological mitochondrial Ca^2+^ overload. However, several challenges remain before these strategies can be considered therapeutically viable. The blood–brain barrier permeability, target selectivity, and long-term safety of candidate interventions have not been evaluated. In addition, because DISC1 and RFX1 also regulate other genes and cellular processes, systemic or long-term targeting of this pathway may cause unintended effects. Therefore, future studies are needed to determine whether pharmacological modulation of this axis can achieve meaningful neuroprotection in vivo, particularly in models of diabetes-associated cognitive impairment.

Several limitations should be considered when interpreting these results. Firstly, although we have demonstrated that S285 phosphorylation of DISC1 mediates its nuclear localization, the precise mechanism by which phosphorylation influences the NLS or the nuclear export signal remains unclear. Future research should employ techniques such as cryo-electron microscopy to determine how phosphorylation alters DISC1’s structure to promote its nuclear translocation. Secondly, while our study has revealed that Pak2 regulates DISC1 phosphorylation and nuclear translocation, how high glucose activates PAK2 remains to be elucidated. Elucidating the specific pathways by which high glucose activates PAK2 would further clarify the pathogenesis of DE and deepen our understanding of the disease, which remains under investigation. Thirdly, endogenous DISC1 phosphorylation at S285 has not yet been validated using phospho-specific antibodies or in vivo functional assays. Furthermore, the pharmacological specificity of the CDC42/RAC1 inhibitor remains to be more definitively established. Fifth, mitochondrial Ca^2+^ overload in the diabetic brain was inferred from convergent indirect measures and direct in vitro recordings, rather than being demonstrated directly in vivo.

## 5. Conclusions

Taken together, our study indicates that the “dual effect” of DISC1 mediates high-glucose-induced neuronal mitochondrial Ca^2+^ overload in DE. Our data emphasize the transcriptional regulatory role of DISC1 and the intricate mechanisms that govern its function. These findings enhance our understanding of DISC1’s functional repertoire and of DE pathogenesis, and they identify the PAK2-DISC1-RFX1-GRP75 axis as a candidate for therapeutic exploration.

## Data Availability

The mass spectrometry proteomics data generated in this study are available within the article and its Supplementary Materials. The ChIP-seq data supporting the findings of this study are openly available in the Cistrome Database at http://cistrome.org/db/#/ (accessed on 1 March 2026), under accession numbers GSM594584 and GSM3318132.

## Supplementary Materials

The following supporting information can be downloaded at https://www.mdpi.com/article/10.3390/biom16091366/s1, Figure S1: Serine 284 and serine 285 were identified as potential phosphorylation sites of DISC1; Figure S2: Preventing the phosphorylation of DISC1 at S285 protects against high glucose driven nuclear translocation of DISC1; Figure S3: High glucose promotes Grp75 expression through the PAK2–DISC1–RFX1 axis in primary neurons; Figure S4: Knockdown of Disc1 leads to mitochondrial Ca^2+^ overload and neuronal damage; Figure S5: Overexpression of Disc1 fails to ameliorate high glucose-induced mitochondrial Ca^2+^ overload in neurons; The original western blot images can be found in Supplementary materials.

## Abbreviations

ChIP	chromatin immunoprecipitation
DE	Diabetic encephalopathy
DISC1	Disrupted in Schizophrenia 1
ER	endoplasmic reticulum
GRP75	Glucose-Regulated Protein 75
IP3R	Inositol 1,4,5-trisphosphate receptor
LEF1	lymphoid enhancer binding factor 1
MAM	Mitochondria-Associated Membrane
MS	Mass Spectrometry
PAK2	P21 (RAC1) activated kinase 2
RFX1	regulatory factor X1
TCA	tricarboxylic acid
VDAC1	Voltage-Dependent Anion Channel 1
