# Supplementary material for: DISC1 Orchestrates Mitochondrial Calcium Overload in Diabetic Encephalopathy Through a Dual Nucleocytoplasmic Mechanism
Source: Biomolecules. 2026 Sep 20;16(9):1366. doi: 10.3390/biom16091366 (PMC13604470; doi:10.3390/biom16091366)

Figure 1K original WB images

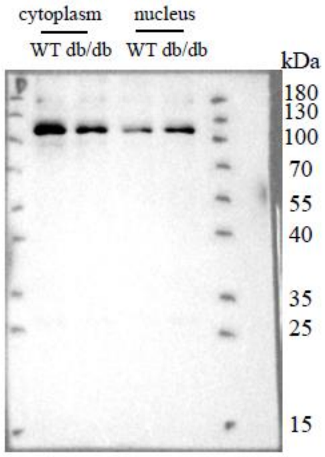

DISC1

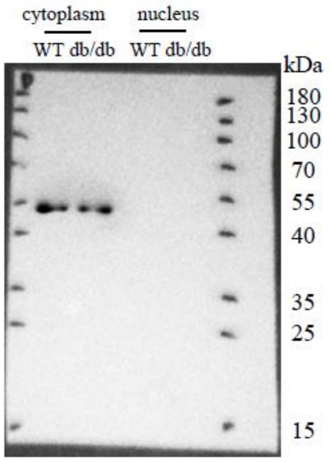

TUBULIN (DISC1)

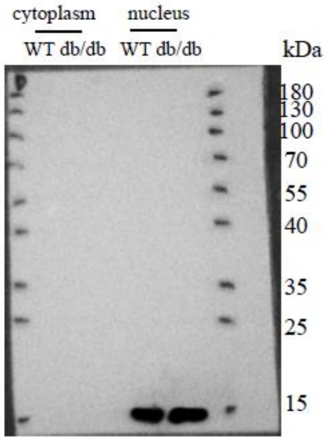

H3 (DISC1)

Figure 2D original WB images

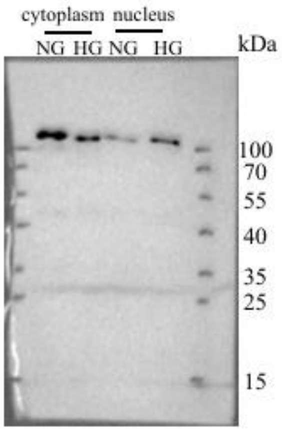

DISC1

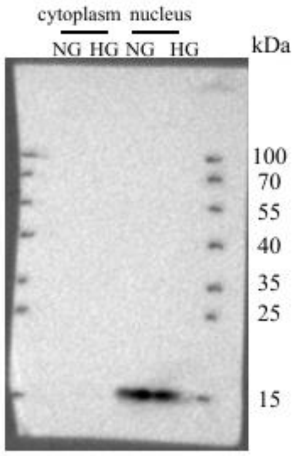

H3 (DISC1)

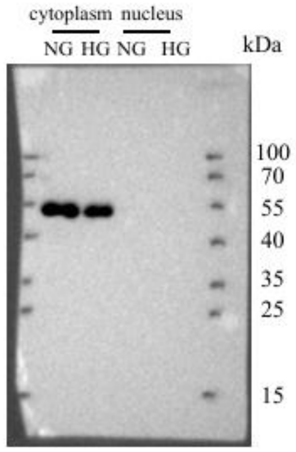

TUBULIN (DISC1)

Figure 3C,D original WB images

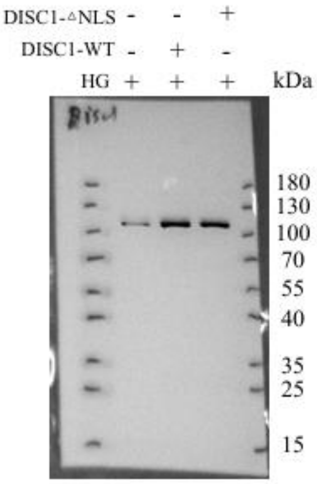

DISC1

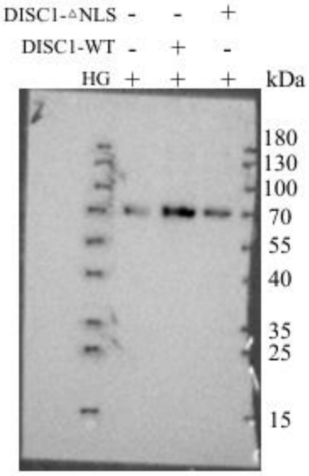

GRP75

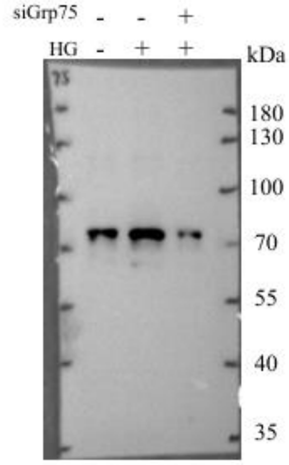

GRP75

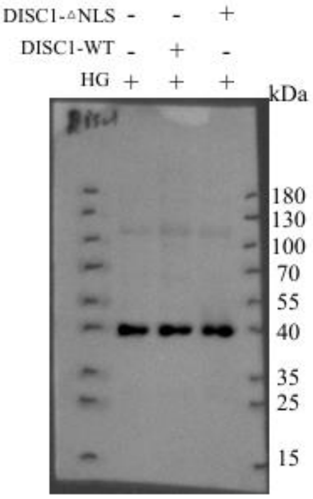

ACTIN (DISC1)

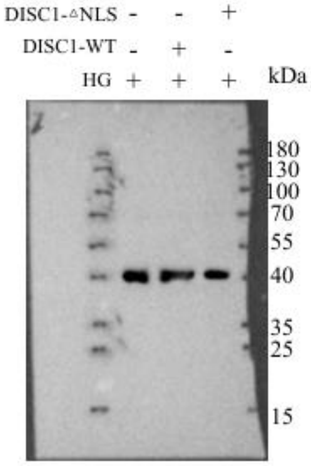

ACTIN (GRP75)

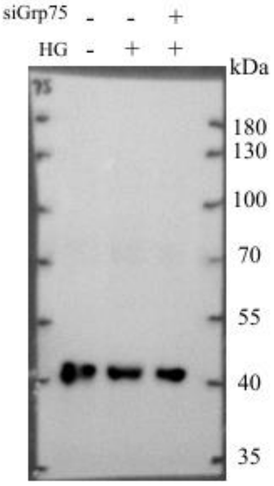

ACTIN (GRP75)

Figure 4A original WB images

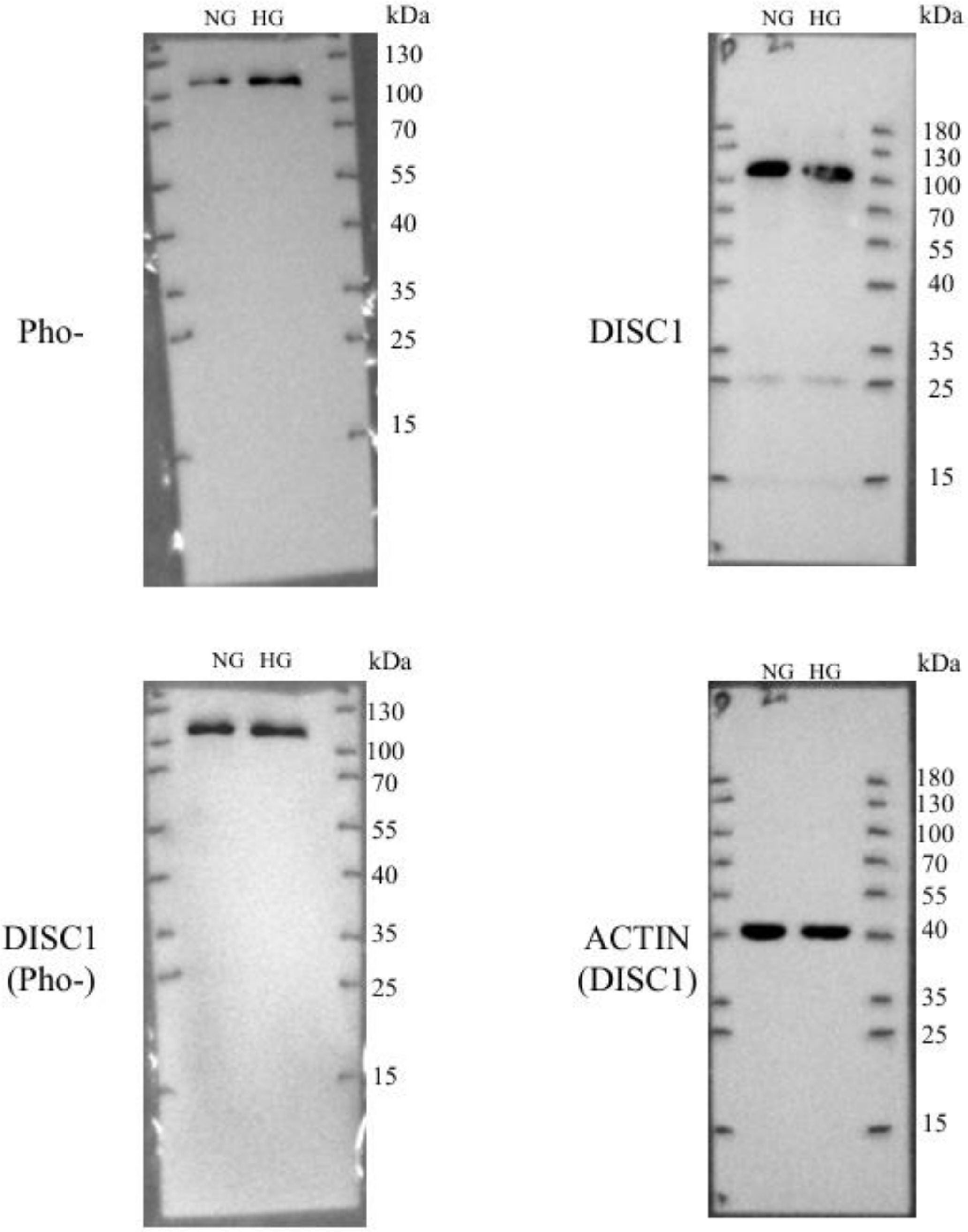

Figure 4C original WB images

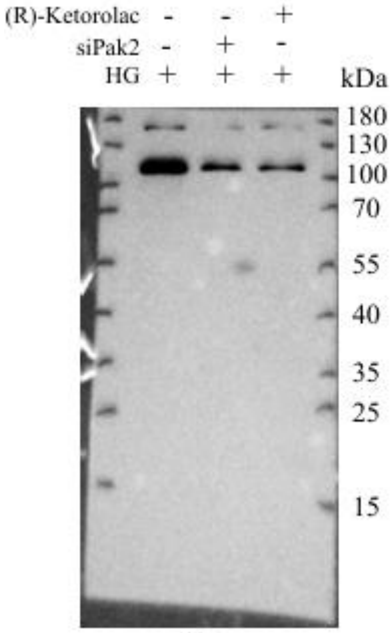

Pho-

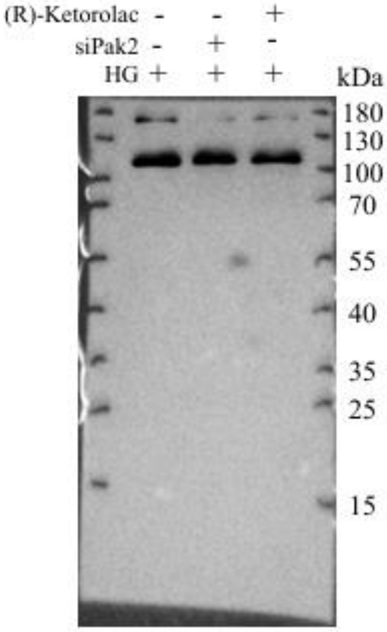

DISC1 (Pho-)

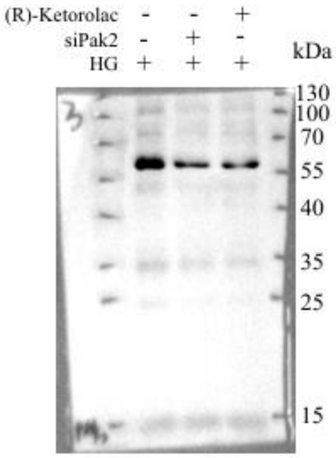

PAK2

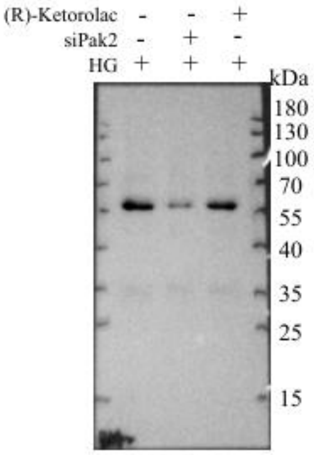

PAK2

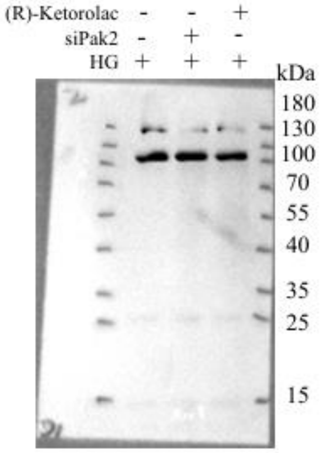

DISC1

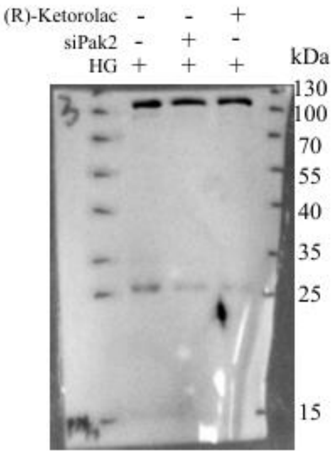

DISC1 (PAK2)

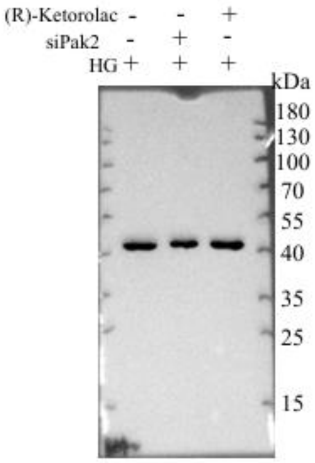

ACTIN (PAK2)

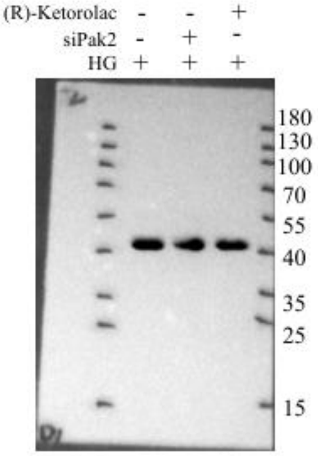

ACTIN (DISC1)

Figure 4D-G original WB images

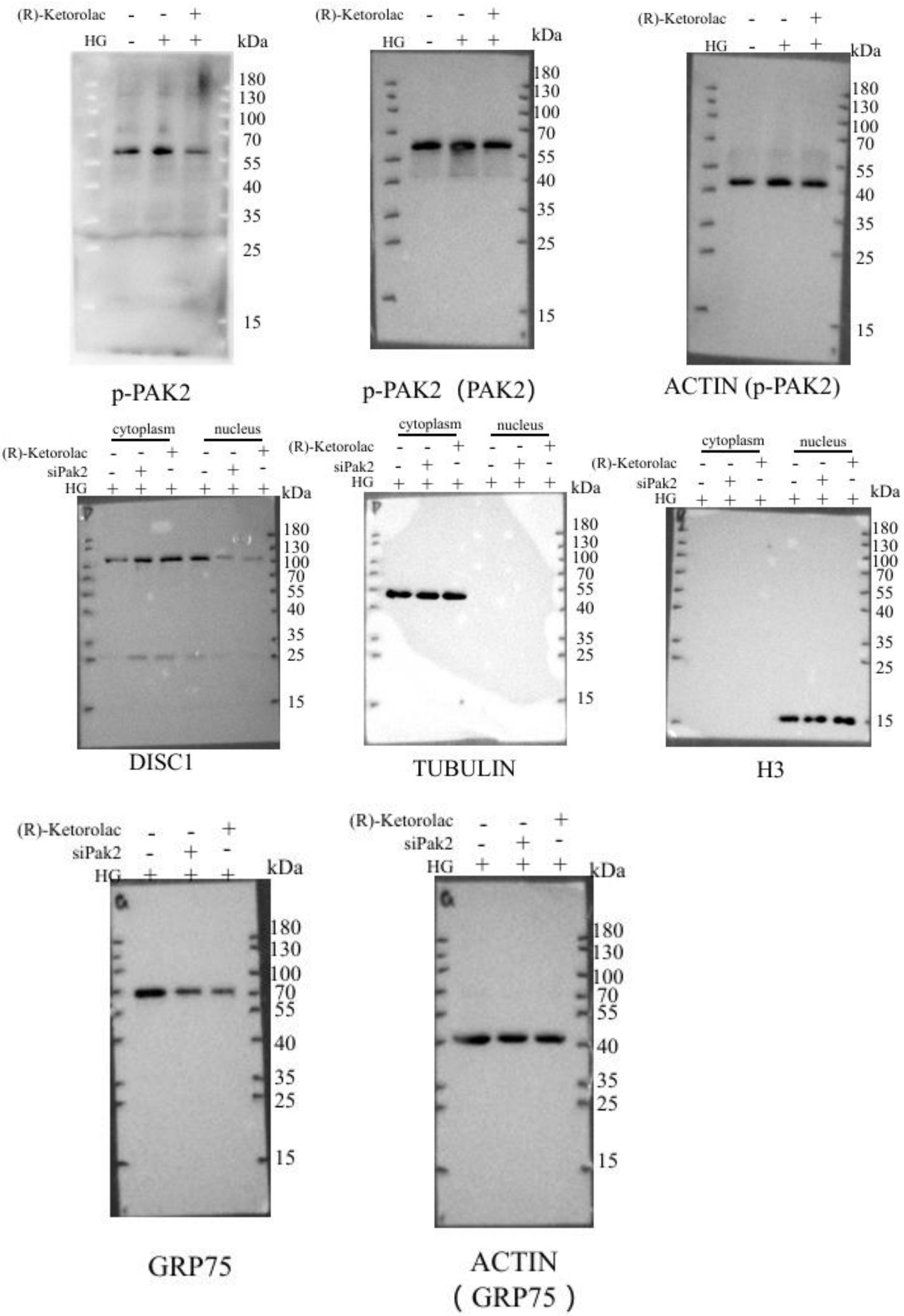

Figure 5A,C original WB images

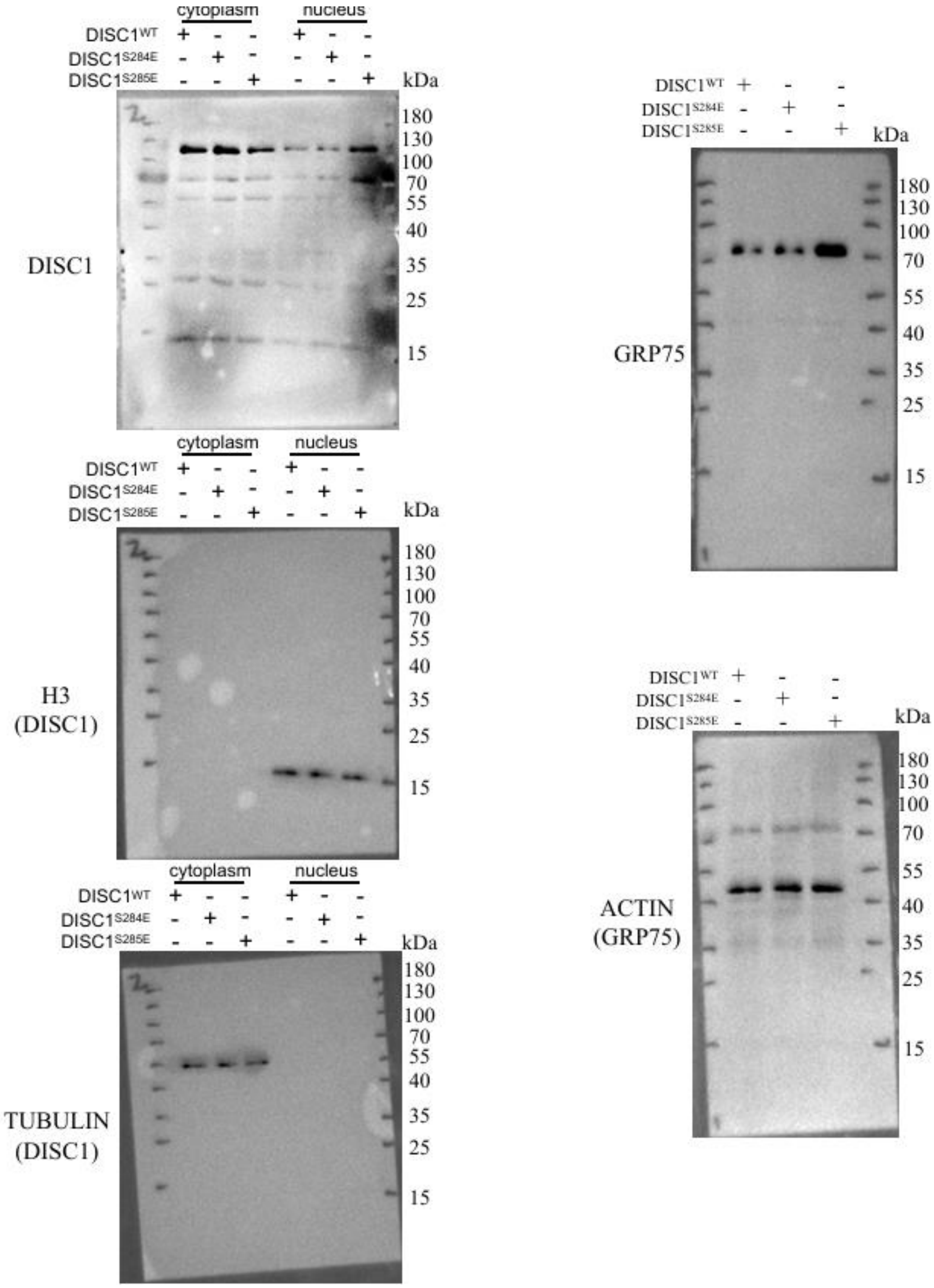

Figure 6D original WB images

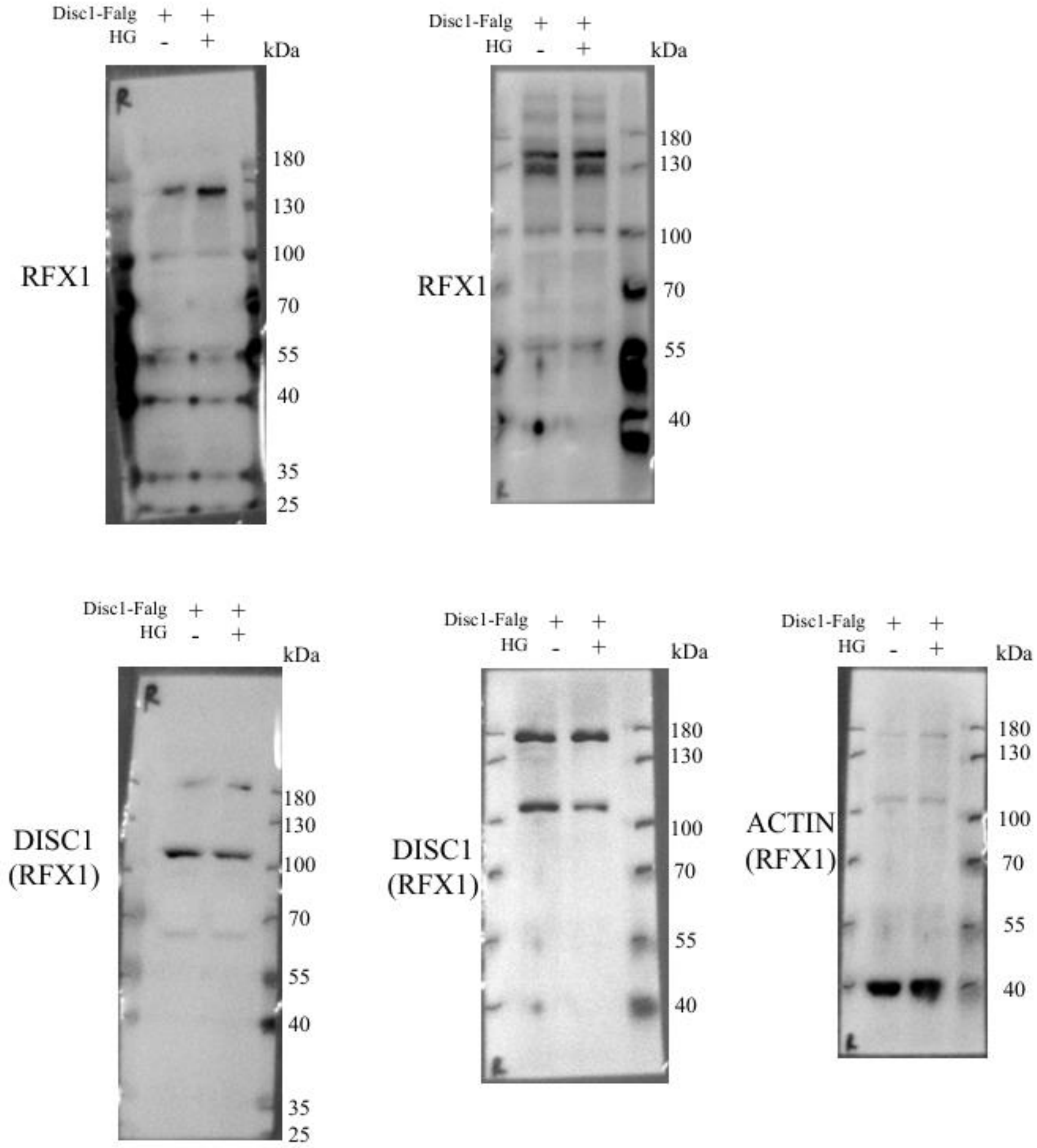

Figure 6G,H original WB images

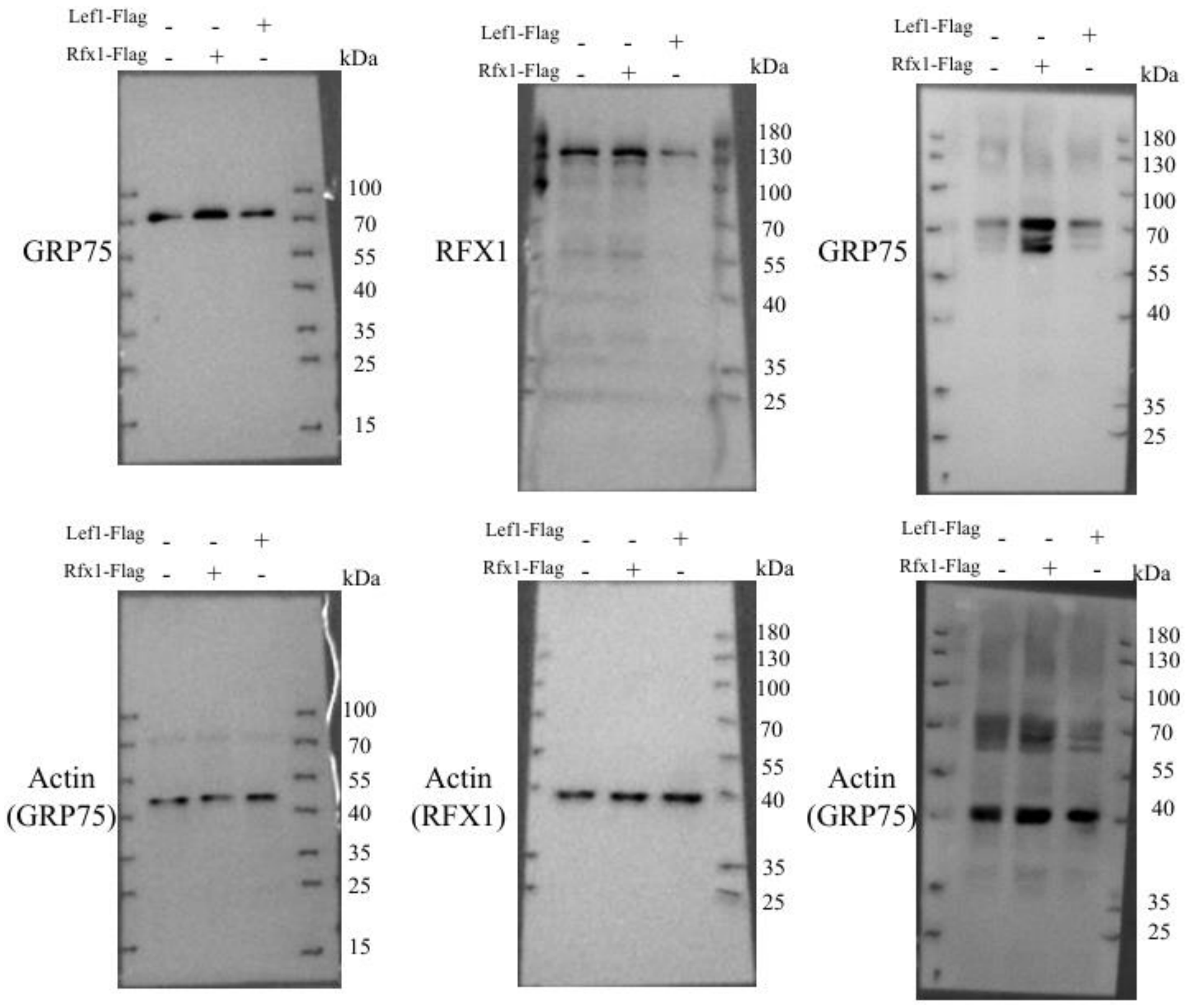

Figure 7A original WB images

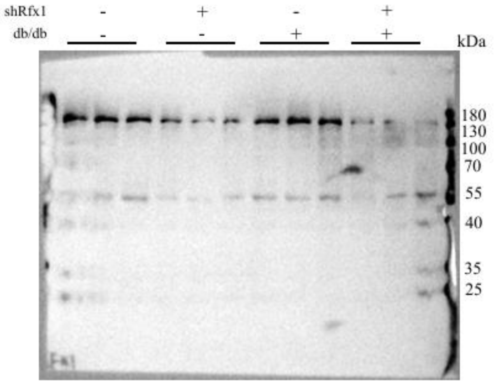

RFX1

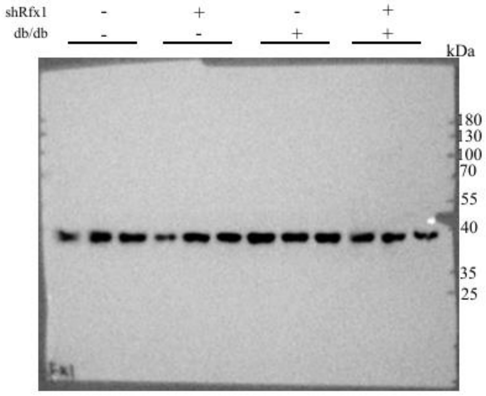

ACTIN
(RFX1)

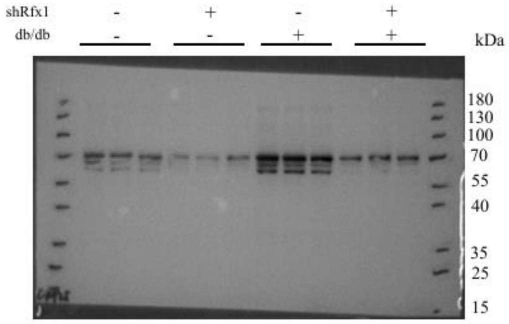

GRP75

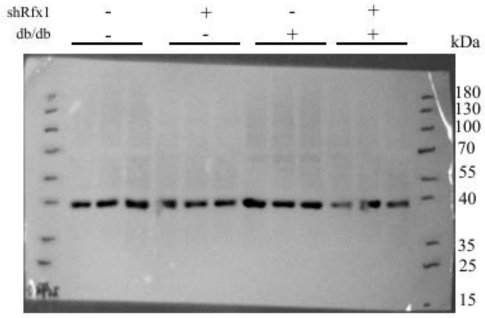

ACTIN
(GRP75)

Figure 8C original WB images

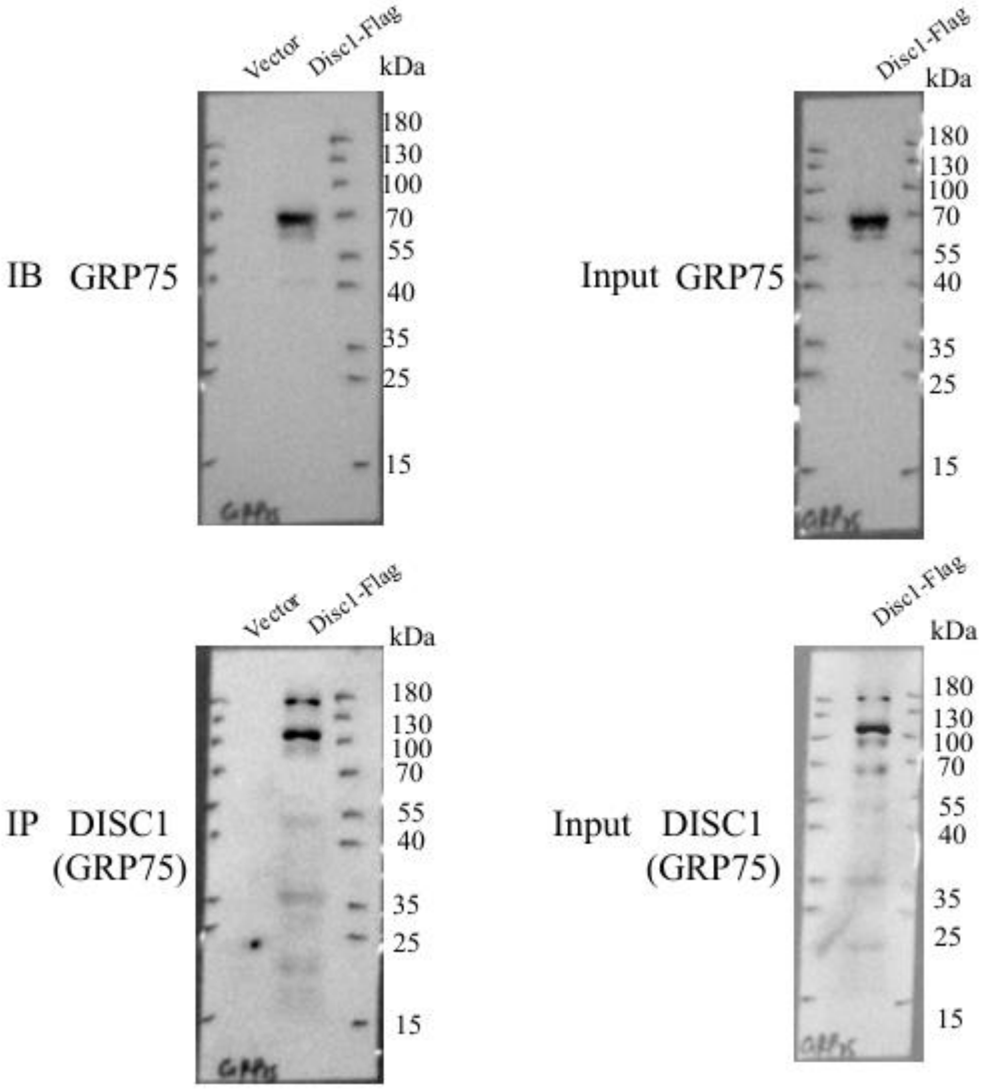

Figure 8D original WB images

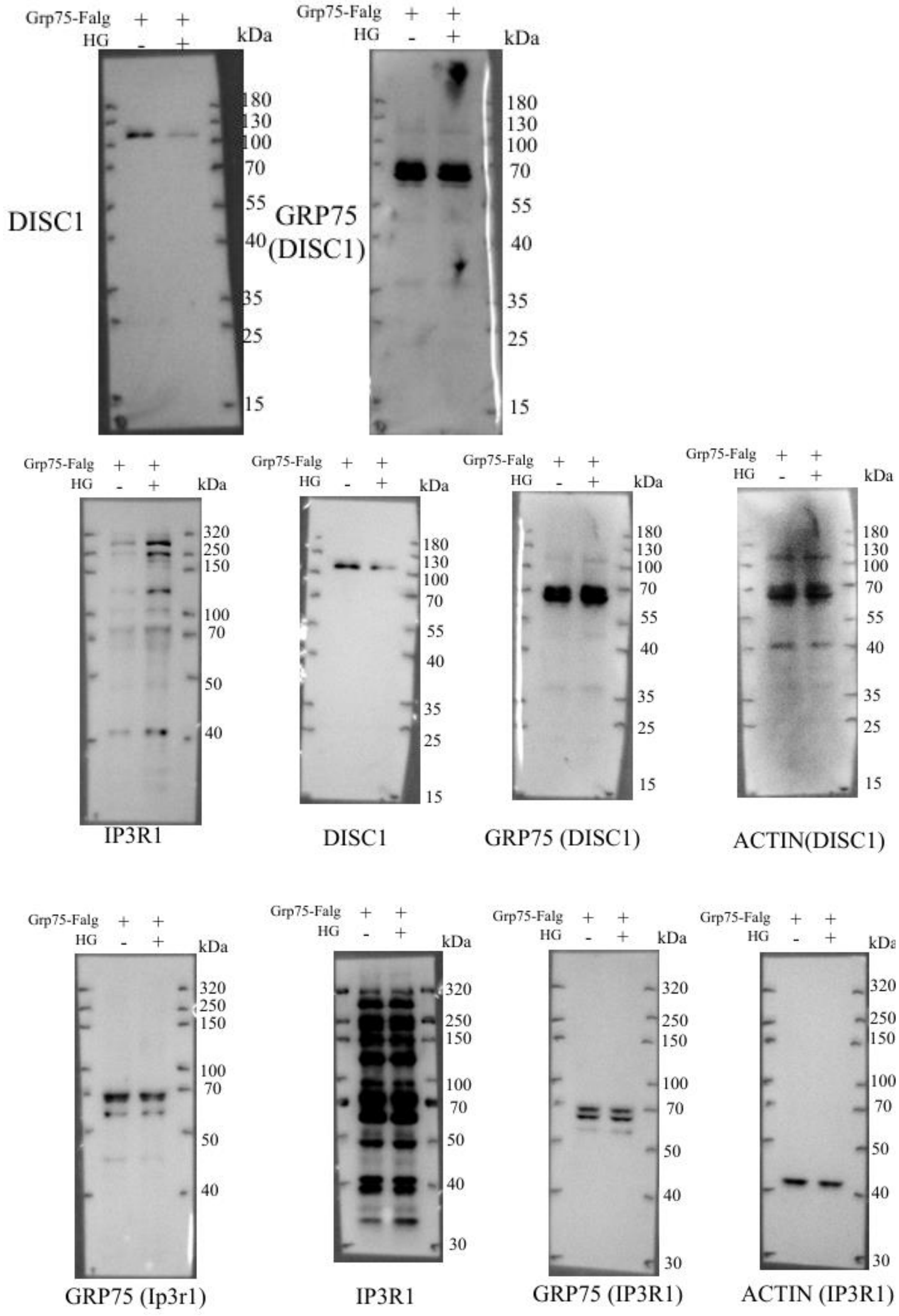

Figure 8F original WB images

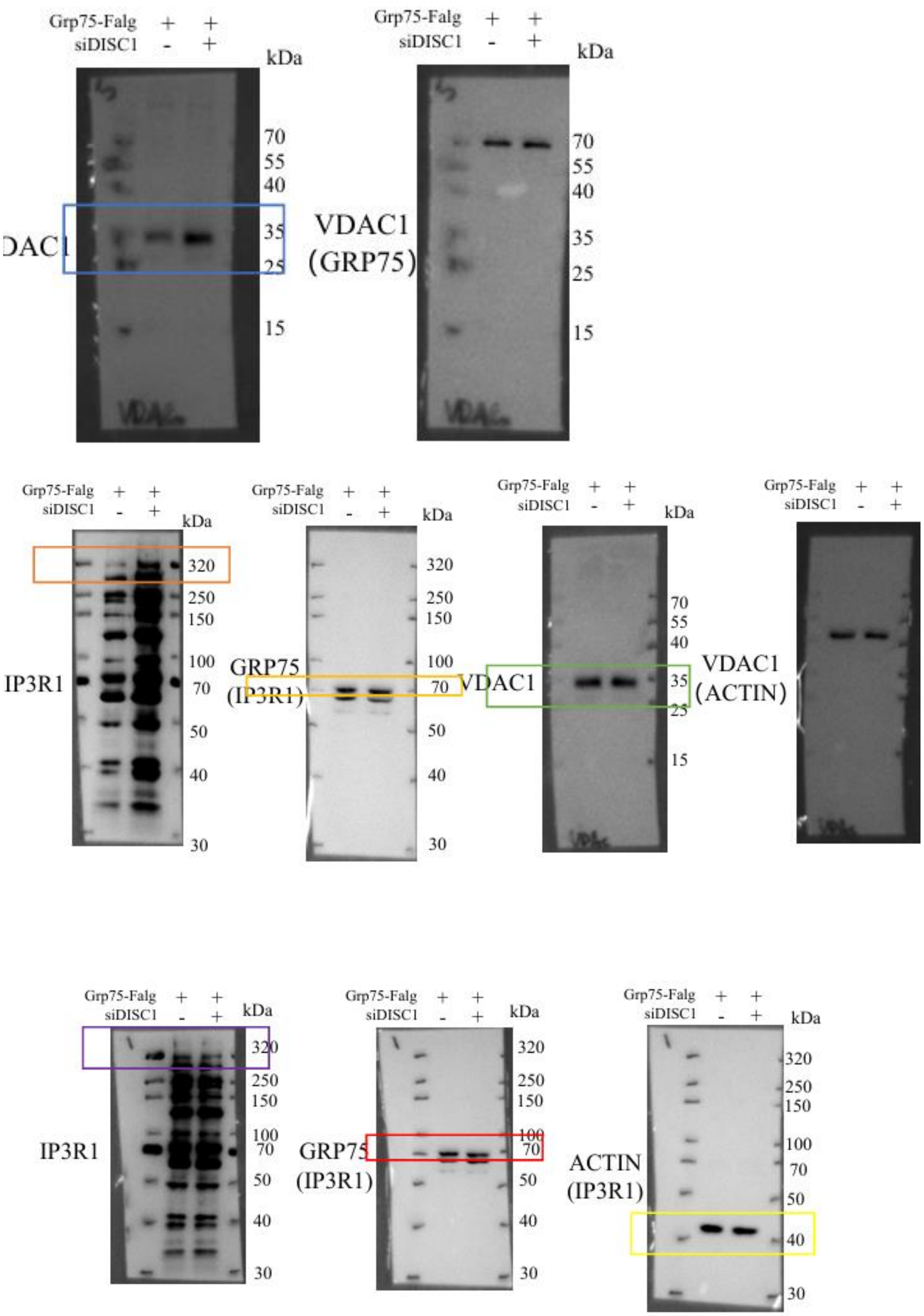

Figure S1A original WB images

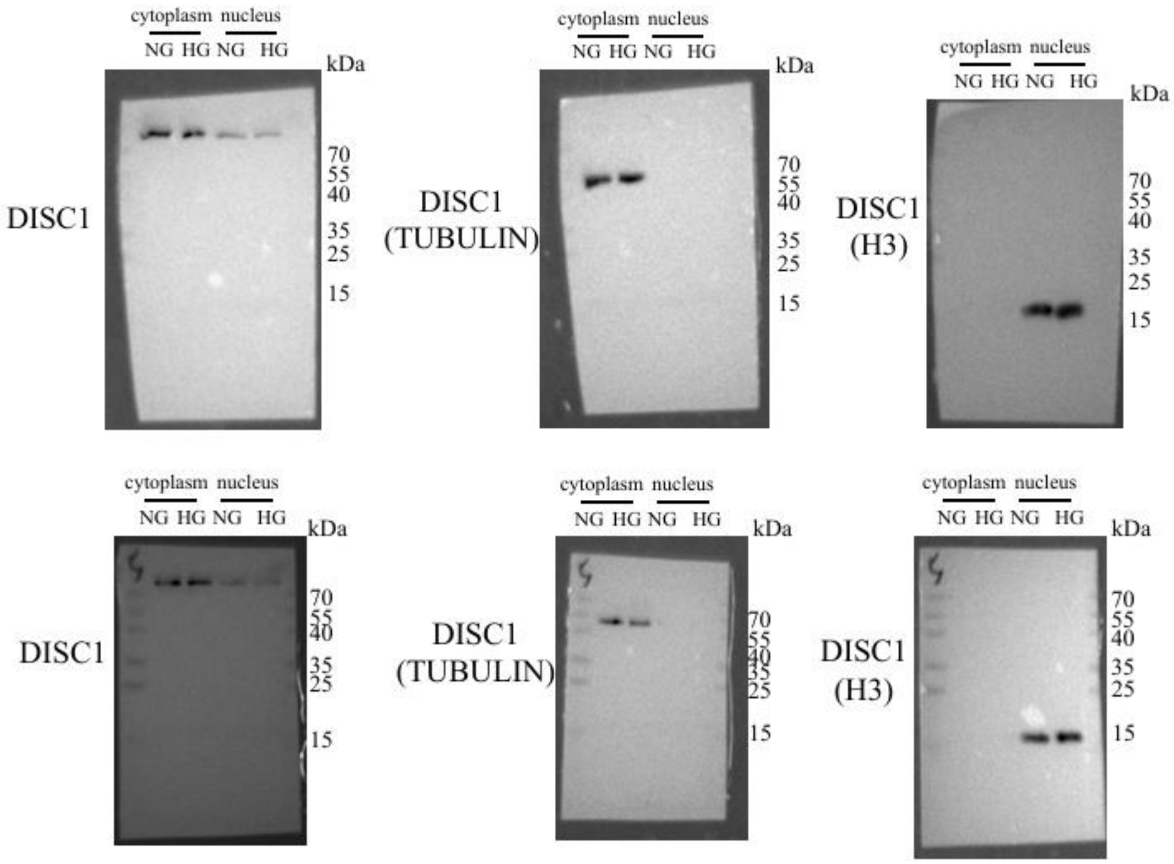

Figure S1F original WB images

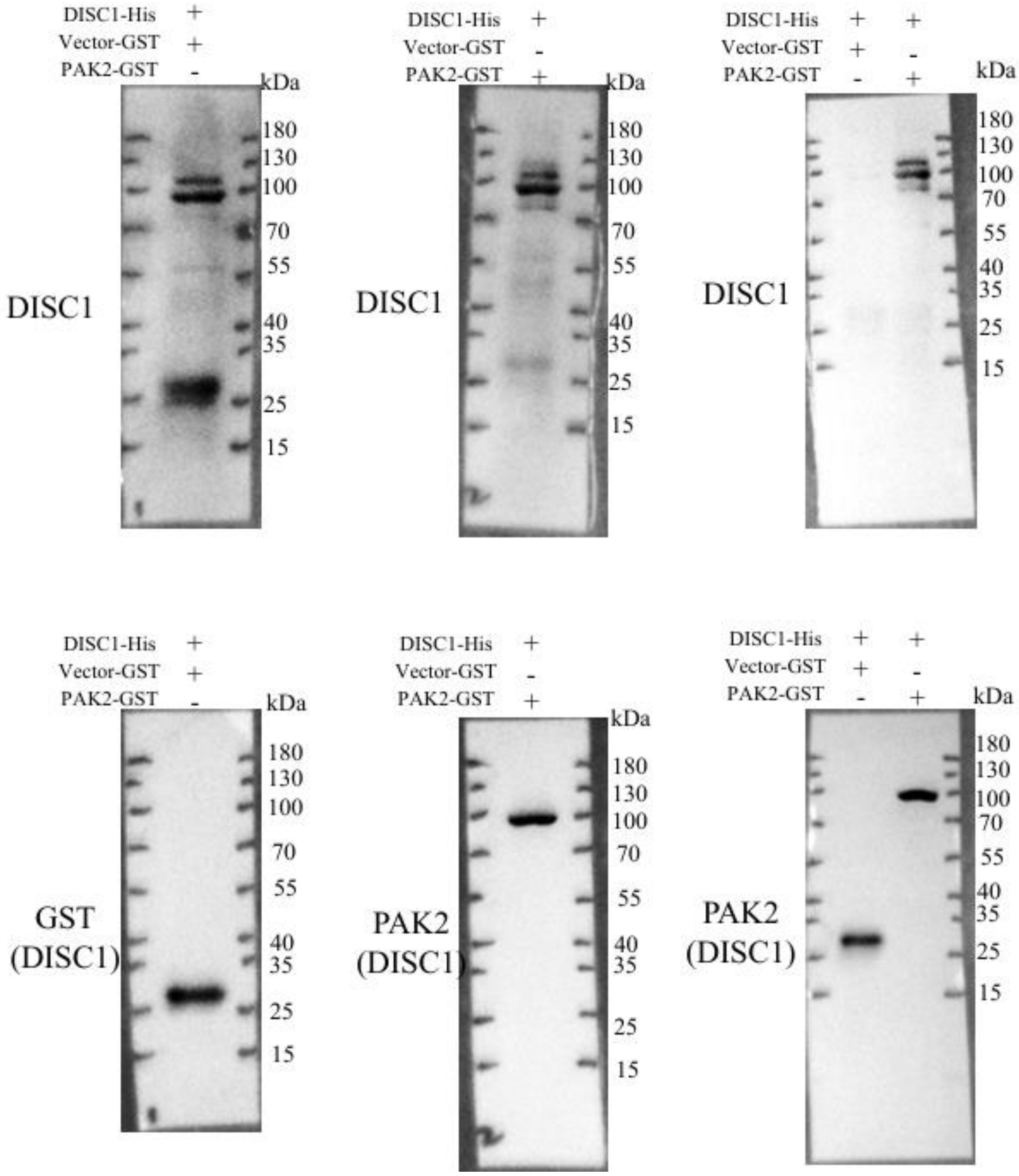

Figure S1G original WB images

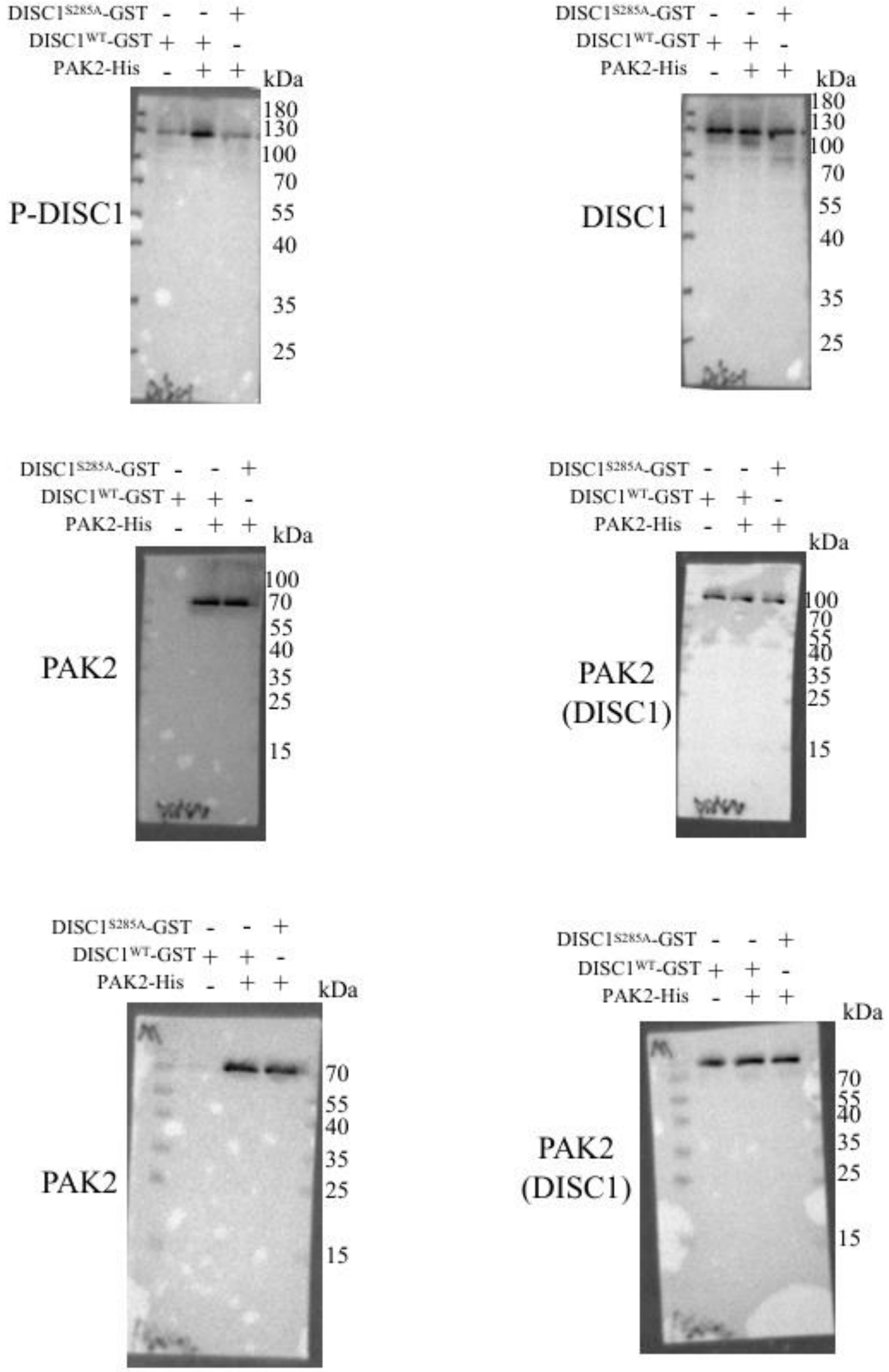

Figure S2A,C original WB images

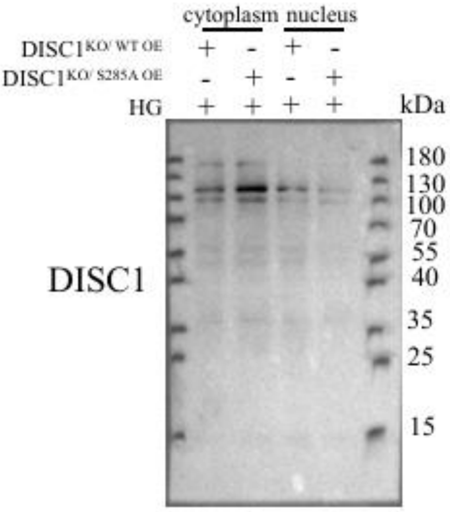

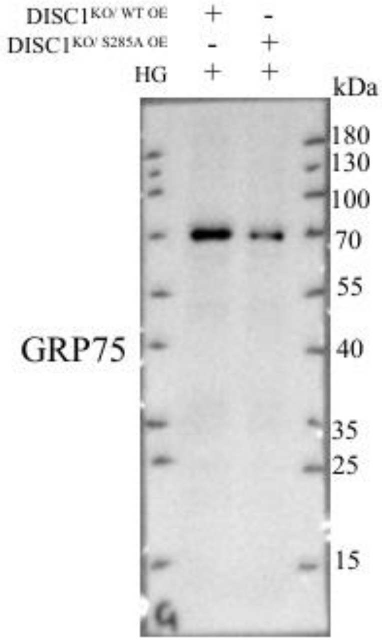

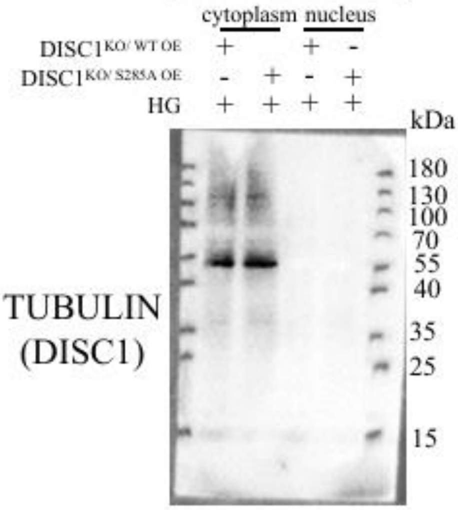

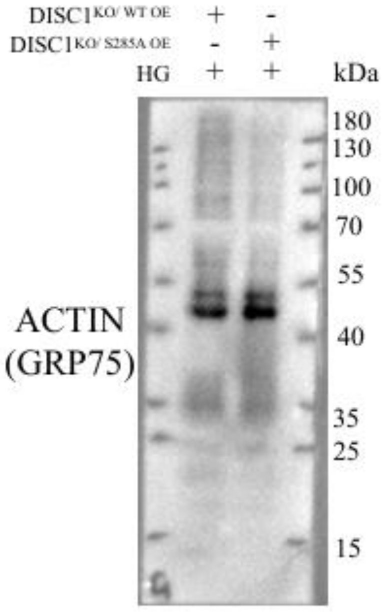

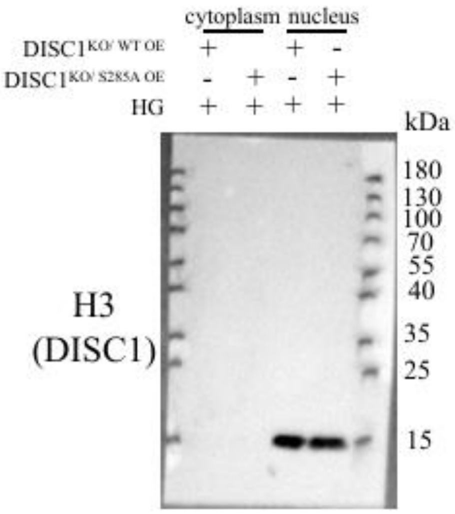

Figure S3A original WB images

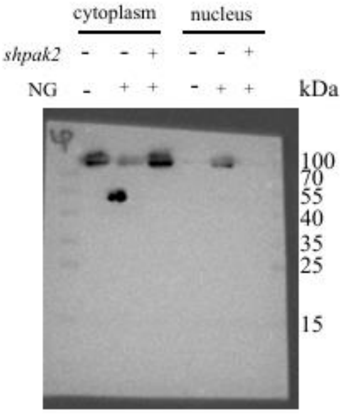

DISC1

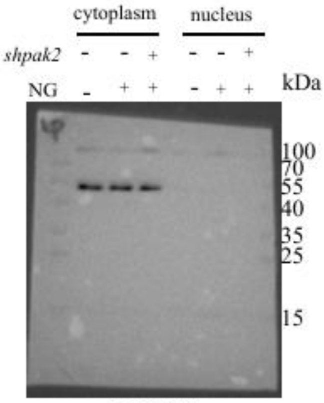

DISC1
(TUBULIN)

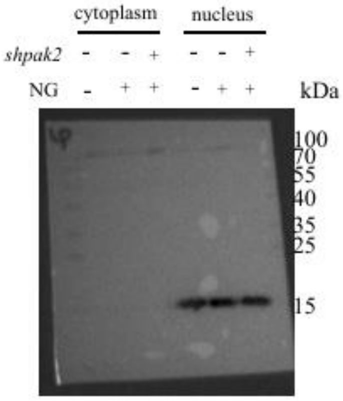

DISC1
(H3)

Figure S3B original WB images

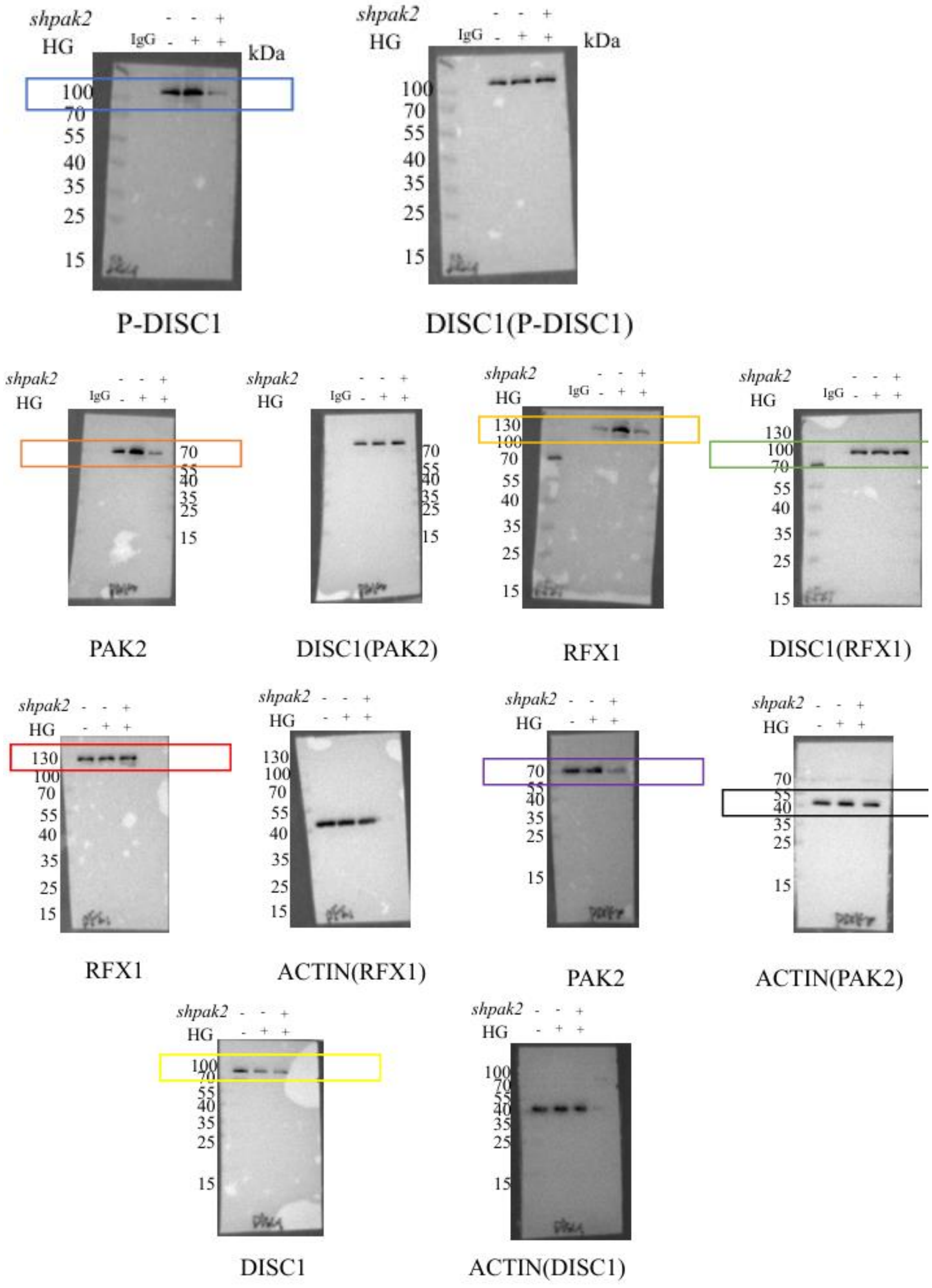

Figure S4A original WB images

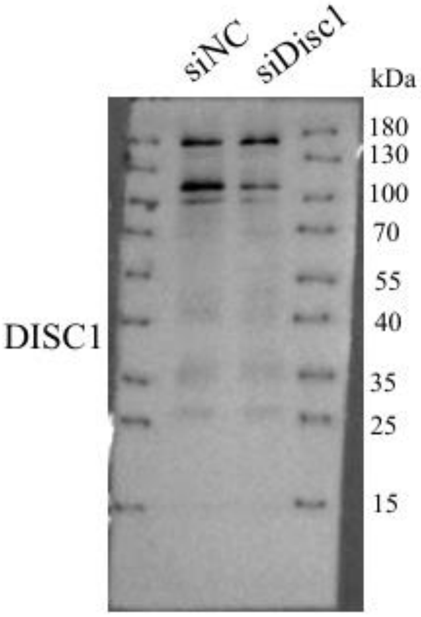

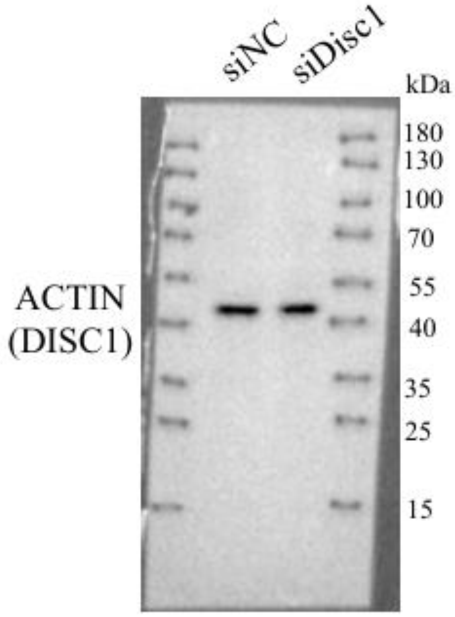

Supplement: Supplementary file 1 [file biomolecules-16-01366-s001.zip › Original WB Images.pdf]
