# Supplementary material for: DISC1 Orchestrates Mitochondrial Calcium Overload in Diabetic Encephalopathy Through a Dual Nucleocytoplasmic Mechanism
Source: Biomolecules. 2026 Sep 20;16(9):1366. doi: 10.3390/biom16091366 (PMC13604470; doi:10.3390/biom16091366)
Supplement: Supplementary file 1 [file biomolecules-16-01366-s001.zip › Supplementary Figures.pdf]

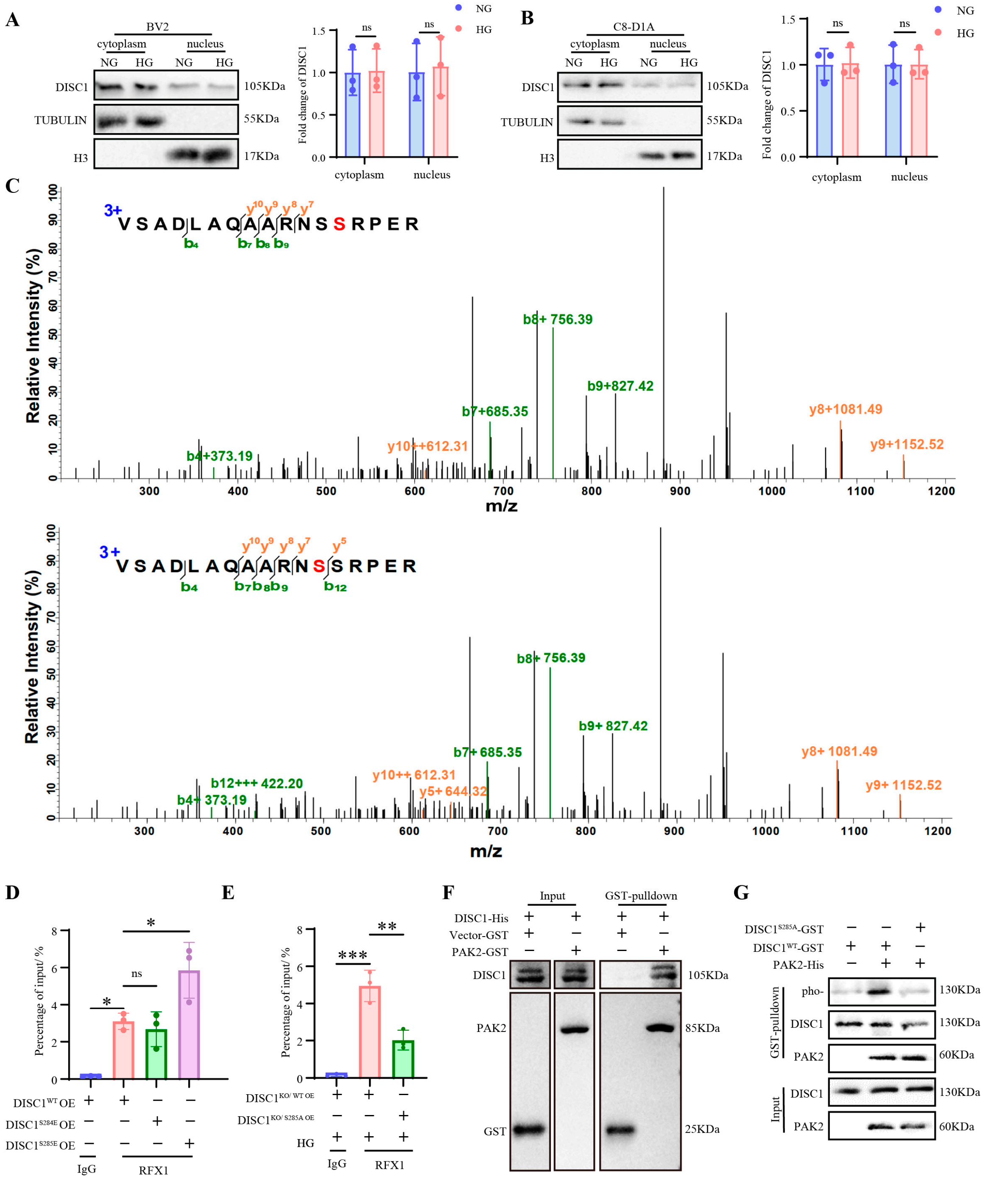

Figure S1 Serine 284 and serine 285 were identified as potential phosphorylation sites of DISC1.

(A) Protein levels of cytoplasmic and nuclear DISC1 in BV2 cells under high-glucose treatment ($n = 3$ independent cultures); **(B)** Protein levels of cytoplasmic and nuclear DISC1 in C8-D1A cells under high-glucose treatment ($n = 3$ independent cultures); **(C)** Mass spectrometry was performed to identify the phosphorylation sites of DISC1 in SH-SY5Y; **(D)** After overexpression of DISC1^{WT}, DISC1^{S284E}, or DISC1^{S285E}, ChIP-qPCR was performed to detect RFX1 enrichment at the *GRP75* promoter in SH-SY5Y cells; **(E)** Following DISC1 knockdown and subsequent rescue with DISC1^{WT} or DISC1^{S285A}, ChIP-qPCR was performed to assess RFX1 enrichment at the *GRP75* promoter in SH-SY5Y cells under high-glucose conditions; **(F)** GST pull-down assay to detect the direct interaction between DISC1 and PAK2; **(G)** In vitro kinase assay for PAK2-mediated DISC1 phosphorylation. Data are

presented as mean ± SD. p-values were determined using an unpaired two-tailed Student's t-test **(A, B)** and one-way ANOVA followed by Tukey's post hoc test **(D, E).** * $p < 0.05$, ** $p < 0.01$, *** $p < 0.001$, and **** $p < 0.0001$.

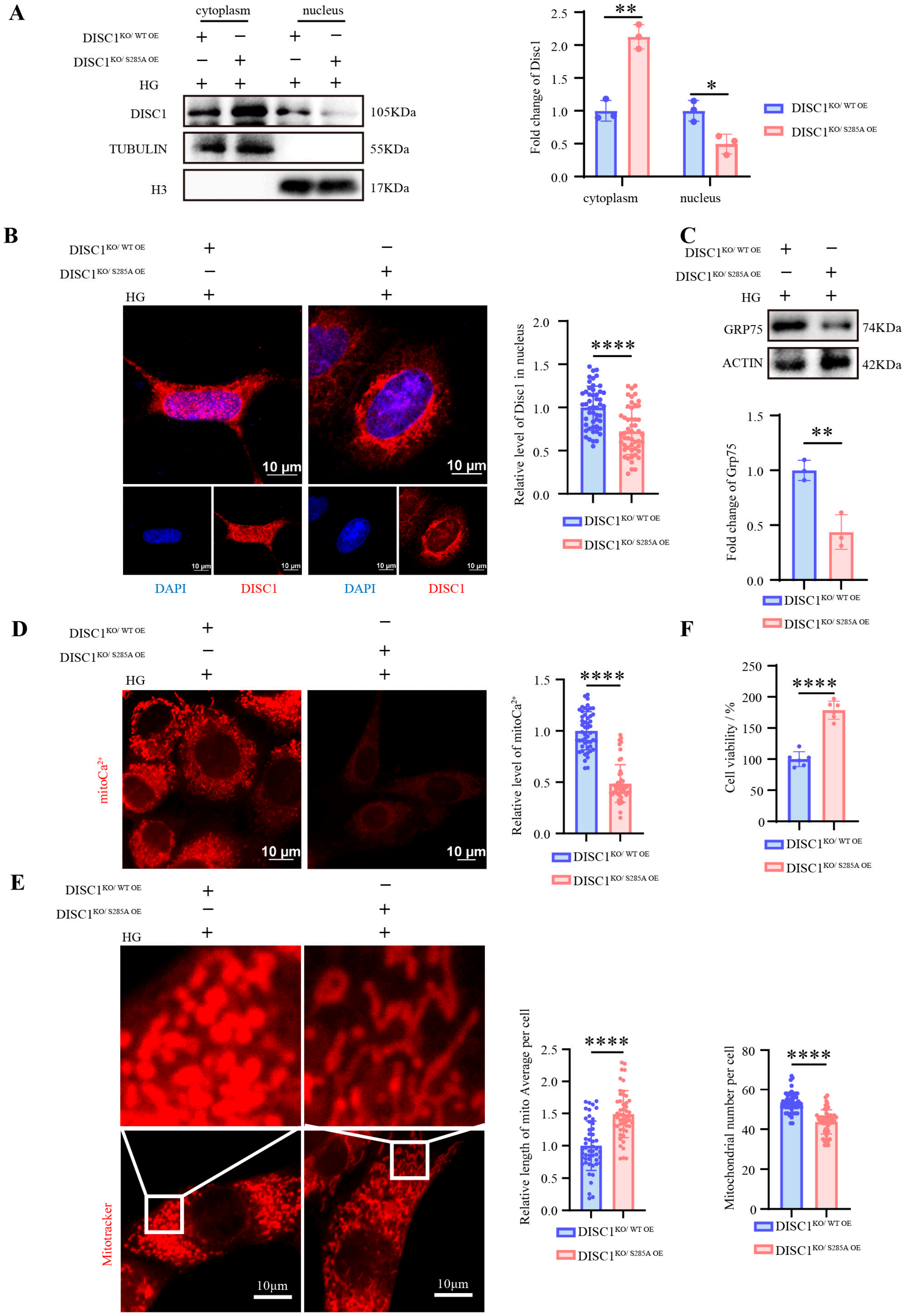

Figure S2 Preventing the phosphorylation of DISC1 at S285 protects against high

glucose-driven nuclear translocation of DISC1.

(A) After DISC1 knockdown, rescue with wild-type DISC1 or the S285A mutant (serine 285 to alanine), western blot was performed to detect cytoplasmic and nuclear DISC1 levels in high-glucose-treated SH-SY5Y cells (n = 3 independent cultures); **(B)** DISC1 localization was examined by immunofluorescence (n = 50 cells from 5 independent cultures); **(C)** GRP75 levels were detected by western blot (n = 3 independent cultures); **(D)** Mitochondrial Ca^{2+} levels were measured (n = 50 cells from 5 independent cultures); **(E)** Mitochondrial morphology was assessed (n = 50 cells from 5 independent cultures); **(F)** Cell viability of HT22 cells was evaluated (n = 6 independent cultures). Data are presented as mean ± SD. p-values were determined using a one-way ANOVA followed by Tukey's post hoc test **(A-F).** * $p < 0.05$, ** $p < 0.01$, *** $p < 0.001$, and **** $p < 0.0001$.

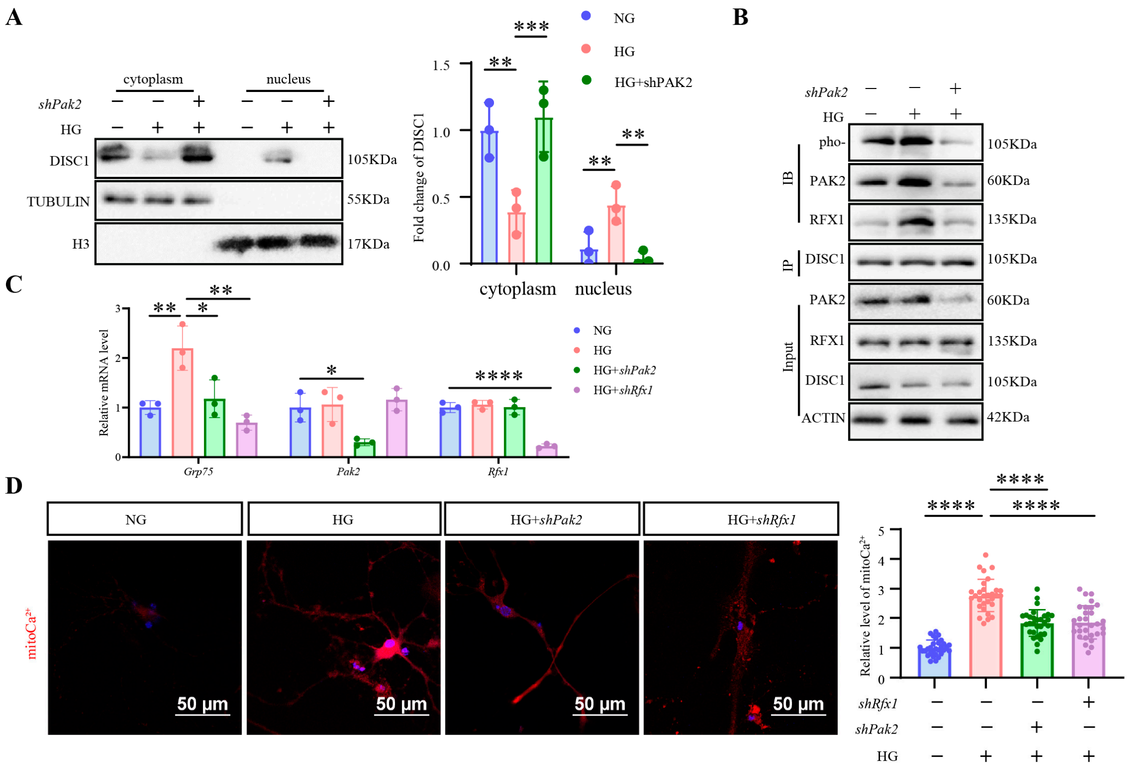

Figure S3 High glucose promotes *Grp75* expression through the PAK2–DISC1–RFX1 axis in primary neurons.

(A) Western blot was performed to detect cytoplasmic and nuclear DISC1 levels in primary neurons (n=3 independent cultures); **(B)** Co-immunoprecipitation followed by detection of DISC1 phosphorylation and the interactions of DISC1 with PAK2 and RFX1; **(C)** qPCR analysis of the mRNA levels of *Grp75*, *Pak2*, and *Rfx1* (n=3 independent cultures); **(D)** Measurement of mitochondrial Ca^{2+} levels using a mitochondrial Ca^{2+}-specific fluorescent probe (n=30 cells from 3 independent cultures). Data are presented as mean ± SD. p-values were determined using a one-way ANOVA followed by Tukey's post hoc test **(A, C, D).** * $p < 0.05$, ** $p < 0.01$, *** $p < 0.001$, and **** $p < 0.0001$.

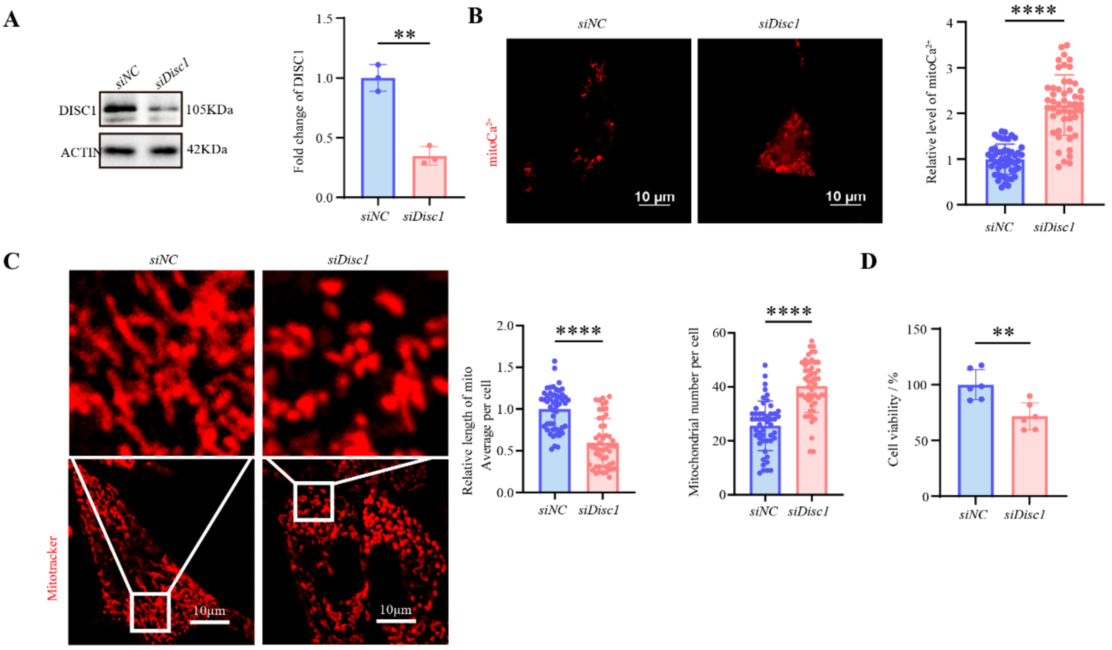

Figure S4 Knockdown of *Disc1* leads to mitochondrial Ca^{2+} overload and neuronal damage.

(A) Western blot validation of *Disc1* knockdown efficiency using siRNA (n=3 independent cultures); **(B)** Mitochondrial morphology was assessed (n=50 cells from 5 independent cultures); **(C)** Measurement of mitochondrial Ca^{2+} levels in HT22 cells using a mitochondrial Ca^{2+}-specific fluorescent probe (n=50 cells from 5 independent cultures); **(D)** Cell viability evaluation by CCK-8 assay in HT22 cells (n=6 independent cultures). Data are presented as mean ± SD. p-values were determined using an unpaired two-tailed Student's t-test **(A-C)** and one-way ANOVA followed by Tukey's post hoc test **(B-D).** * $p < 0.05$, ** $p < 0.01$, *** $p < 0.001$, and **** $p < 0.0001$.

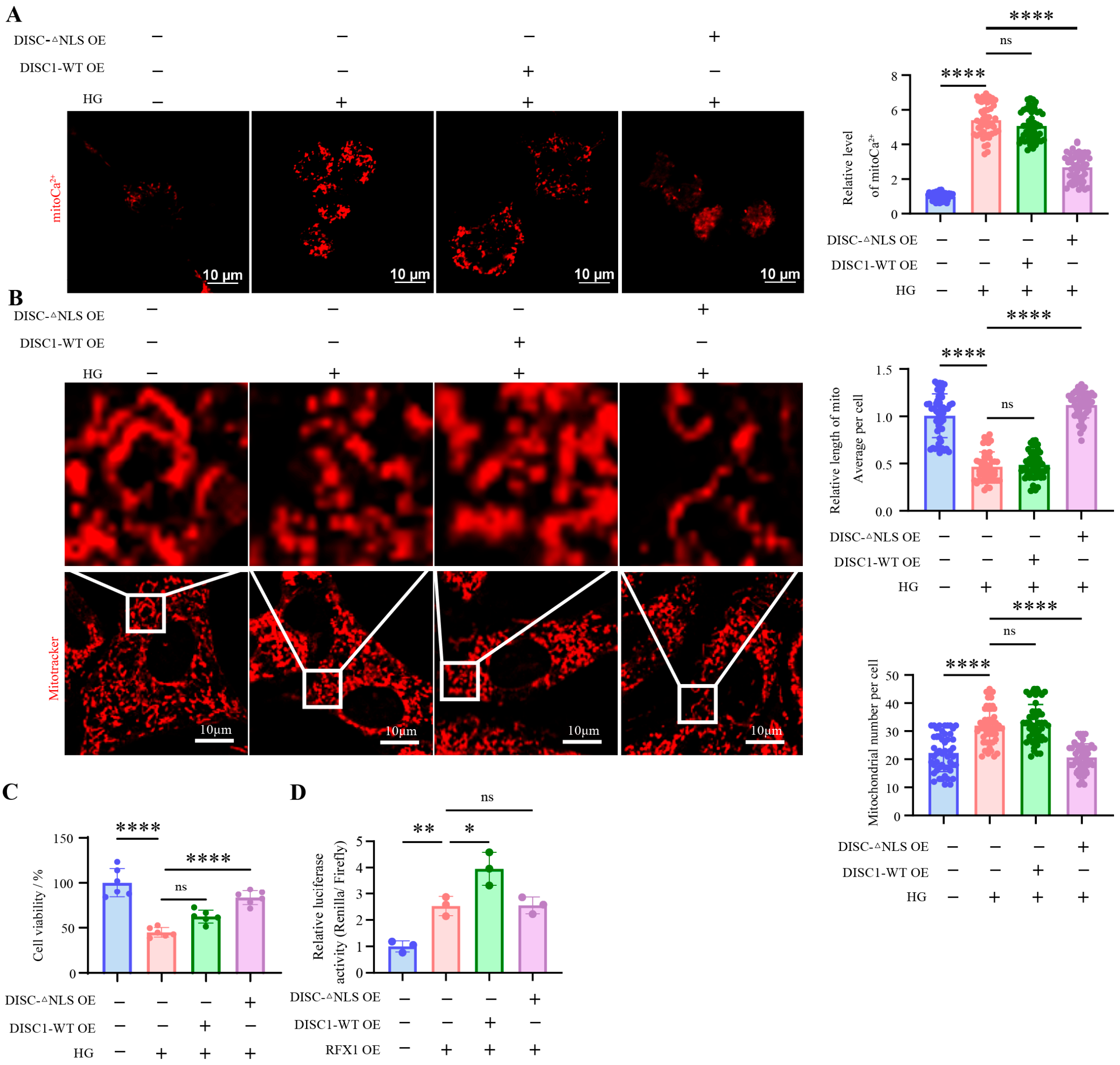

Figure S5 Overexpression of Disc1 fails to ameliorate high glucose-induced mitochondrial Ca^{2+} overload in neurons.

(A) After overexpression of wild-type DISC1 or NLS-deleted DISC1, mitochondrial morphology was assessed in SH-SY5Y cells treated with high glucose (n=50 cells from 5 independent cultures); **(B)** Measurement of mitochondrial Ca^{2+} levels in SH-SY5Y cells using a calcium-specific fluorescent probe (n=50 cells from 5 independent cultures); **(C)** Cell viability evaluation by CCK-8 assay in SH-SY5Y cells (n=6 independent cultures); **(D)** Luciferase reporter assays were performed to evaluate the regulatory effect of RFX1 on genes driven by the Grp75 promoter (n=3 independent cultures). Data are presented as mean ± SD. p-values were determined using an unpaired two-tailed Student's t-test **(A-C).** * $p < 0.05$, ** $p < 0.01$, *** $p < 0.001$, and **** $p < 0.0001$.
